# Polymorphism, gigantism, and cannibalism, one stylonychid ciliate (Ciliophora, Hypotricha) to rule them all

**DOI:** 10.3389/fmicb.2023.1159634

**Published:** 2023-05-17

**Authors:** Atef Omar, Seok Won Jang, Jae-Ho Jung

**Affiliations:** ^1^Natural Science Research Institute, Gangneung-Wonju National University, Gangneung, Republic of Korea; ^2^Protist Research Team, Nakdonggang National Institute of Biological Resources, Sangju, Republic of Korea; ^3^Department of Biology, Gangneung-Wonju National University, Gangneung, Republic of Korea

**Keywords:** morphology, ontogenesis, phylogeny, *Tetmemena indica* nov. stat., *Tetmemena polymorpha* n. sp.

## Abstract

The morphology, ontogenesis, and molecular phylogeny of the polymorphic and cannibalistic giant forming *Tetmemena polymorpha* n. sp., found in a brackish water sample in South Korea, were investigated. The present species has long been misidentified as “*Oxytricha bifaria*.” The new investigation shows that the species produces three morphologically different morphs. The small morph is bacterivorous and characterized by its small body size and slim body and it is found only in the stationary and decline phases of the culture. The large morph has a wide body, larger oral apparatus, and feeds on small eukaryotes such as yeast cells and small ciliates. It divides very quickly and produces the other two morphs and found in the exponential phase of the cultures. The giant morph is characterized by its huge body and oral apparatus. It feeds on the small morph cells of the same species and other ciliates, and occurs together with the small morph. Phylogenetic analyses based on the 18S rRNA gene sequences show that the new species is placed in a sister subclade to that containing other *Tetmemena* sequences. Moreover, *Tetmemena indica* Bharti et al., 2019 nov. stat. is raised to species level based on the differences in the cyst morphology and the dorsal ciliature to the authoritative *Tetmemena pustulata* population.

## Introduction

The genus *Tetmemena* Eigner, 1999 is a stylonychid group of ciliates commonly found in freshwater, with the most known member, *Tetmemena pustulata* (Müller, 1786) Eigner, 1999 (type species), having a worldwide distribution and was also reported from marine, terrestrial, and sewage habitats (Berger, 1999). Originally, *Tetmemena* was established for stylonychid species with anlagen IV–VI of the proter originated from the frontoventral cirrus IV/3. To date, two species, two subspecies, and one invalid species have been classified in the genus *Tetmemena* (Eigner, 1999; Berger, 2001; Kumar et al., 2016; Bharti et al., 2019; Gupta et al., 2020). For many years, several stylonychid populations were assigned to *T. pustulata* mainly because they possess 18 frontal-ventral-transverse cirri and their transverse cirri are arranged in a single group. Furthermore, there was an underestimation of other characteristics, such as the resting cyst morphology and the dorsal ciliature, as only population-dependent variations (Berger, 1999; Bharti et al., 2019; Kaur et al., 2020). The formation of cannibalistic giants is a very rare and unusual character within the hypotrich ciliates. It was known from very few taxa including an Italian stylonychid population misidentified as “*Oxytricha bifaria*”, now *Tetmemena bifaria*, which is characterized by a different arrangement of the transverse cirri, i.e., in two groups. The Italian population was the subject of more than 60 investigations in the period from 1975 to 1998 as listed by Berger (1999). However, none of these studies dealt with the taxonomic assignment of the population (Esposito and Ricci, 1975; Ricci et al., 1975b, 1980, 1998; Esposito et al., 1976; Banchetti et al., 1978, 1982; Ricci, 1981, 1982). Later, Berger (1999) suggested classifying the Italian population into *Tetmemena pustulata*, but he required further detailed description before the final assignment.

In the present study, we describe a stylonychid ciliate with an unusual lifestyle, i.e., polymorphic, producing three morphologically different individuals (small, large, and giants) that could be mistakenly misidentified as distinct species if observed separately. The cannibalistic giant morph feeds on individuals of the small morph, while the large morph (middle-sized) is only produced when the food source, for instance, bacteria and small eukaryotes, is abundant. The present species is identical to the misidentified population from Italy. Our new morphological and molecular analyses show that it is a distinct species and emphasize the taxonomic value of the polymorphic lifestyle, the cyst morphology, and the dorsal ciliature characters in the classification of the stylonychine ciliates.

## Materials and methods

### Sample collection and identification

A water sample including sediments was collected using a plastic bottle (500 ml) from the estuarine area of Namdaecheon Stream in Gangneung-si (37° 46′ 11″N, 128° 56′ 58″E), South Korea, on 18 April 2022 (Supplementary Figure 1). The water salinity was 4.7‰, and the temperature was 17.1°C as measured in the field using the handheld YSI Pro1030 Water Quality Meter (YSI Inc., Yellow Springs, OH, USA). The water sample was kept in a plant culture dish (SPL Life Sciences, Gyeonggi-do, Korea; 100 mm diameter × 40 mm depth) at room temperature (~20°C). Several clone cultures using single cells of different sizes were established in artificial saline water, with the same salinity as the raw culture, enriched with sterilized wheat grains and baker's yeast as food sources. All clone cultures produced small, large, and giant morphs, and thus, only a single clone culture was kept and used in morphological and molecular analyses. After a few weeks of cultivation in saline water, mineral water (Jeju Samdasoo, Jeju Province Development Co., South Korea) was used successfully to maintain the culture for several months. Some subcultures were fed also on *Dexiostoma* sp. or *Tetrahymena* sp. Living specimens were investigated using a stereomicroscope (Olympus SZ61, Tokyo, Japan) and light microscope (Olympus BX53) with differential interference contrast at magnifications of 50–1,000×. The infraciliature was revealed by protargol impregnation and scanning electron microscopy (SEM). Protargol powder was synthesized using the method of Pan et al. (2013) and Kim and Jung (2017), and the protargol impregnation technique is based on “procedure A” of Foissner (1991, 2014). Cysts were collected from the clone culture or produced by the starvation of large morph specimens. The SEM technique was conducted following the procedure of Foissner (2014) and Moon et al. (2020). Terminology is according to Berger (1999).

### DNA extraction, PCR amplification, and sequencing

Five cells from each morph were collected using a glass micropipette from the clone culture under the stereomicroscope. The cells were transferred to mineral water, starved for about 3 h to allow the digestion of food vacuoles contents in the giant morph specimens, washed at least five times to remove the yeast cells, and then each cell was transferred to a 1.5 ml centrifuge tube with a minimum volume of water. Genomic DNA was extracted using a RED-Extract-N-Amp Tissue PCR Kit (Sigma, St. Louis, MO, USA). The 18S rRNA gene was amplified using the primers New Euk A (Jung and Min, 2009) and LSU rev4 (Sonnenberg et al., 2007) to cover nearly the entire 18S rRNA gene. The PCR conditions were as follows: denaturation at 94°C for 1 min 30 s, followed by 40 cycles of denaturation at 98°C for 10 s, annealing at 58.5°C for 30 s, and extension at 72°C for 3 min, and a final extension step at 72°C for 7 min. For the purification of the PCR products, MEGAquick-spin Total Fragment DNA Purification Kit (iNtRON, South Korea) was used. The sequence fragments determined by the New Euk A primer were identical among all 15 cells; thus, we completed the direct sequencing using one cell from each morph. DNA sequencing was performed using internal primers 18SR300, 18SF790v2, and 18SF1470 (Park et al., 2017; Jung et al., 2018) and an ABI 3700 sequencer (Applied Biosystems, Foster City, CA, USA).

### Phylogenetic analyses

The 18S rRNA gene sequence of *Tetmemena polymorpha* n. sp. was assembled using Geneious 9.1.5 (Kearse et al., 2012). To determine the phylogenetic position of the new species, rRNA gene sequences of 134 hypotrich ciliates were retrieved from the NCBI database including the outgroup taxa, the urostylid clades I and II, and the 35 stylonychids [*Coniculostomum monilata* (MT364889), *Laurentiella strenua* (AJ310487, HM140403, JX893368), *Metastylonychia nodulinucleata* (KY353799), *Pseudostylonychia obliquocaudata* (ON054917), *Stylonychia ammermanni* (FM209295, KP271125, MN159076), *S. koreana* (KX344906), *S. lemnae* (AF164124, AF508773, AJ310496, AJ310497, AM086653, AM086654, AM233913–AM233917, AM260993, AM260994, KX138655, and MN159068), and *S. mytilus* (AF164123, AF508774, AJ310498, AJ310499, AM086663–AM086667, and EF535730)] from Omar et al. (2022).

The sequences were aligned together using ClustalW (Thompson et al., 1994), and both ends were manually trimmed in BioEdit 7.0.9.0 (Hall, 1999). Ambiguous nucleotide alignment positions were masked using Gblocks version 0.91b (Talavera and Castresana, 2007) with less stringent options, such as “allowed gap positions (with half) within the final blocks.” The final alignment showed a final matrix of 1677 columns. jModelTest 2.1.7 (Darriba et al., 2012) was used to select the best-fit model GTR + I + G under the Akaike information criterion (AIC). The maximum likelihood (ML) tree was constructed using IQ-Tree 1.5.3 (Nguyen et al., 2015) with 100,000 bootstrap replicates. The pairwise sequence similarity among taxa was calculated in MEGA 6.06 (Tamura et al., 2013). MrBayes 3.1.2 (Ronquist et al., 2012) was used for Bayesian inference (BI) analyses with Markov chain Monte Carlo (MCMC) for 3,000,000 generations with a sampling frequency of every 100 generations, and the first 7,500 trees were discarded as burn-in. The average standard deviation of split frequencies was 0.0049, the average potential scale reduction factor for parameter values (PSRF) was 1.001, and the effective sample sizes (ESSs) were >200. Phylogenetic trees were visualized using the free software package FigTree ver. 1.4.3 (http://tree.bio.ed.ac.uk/software/figtree/). Bootstrap values ≥95 were considered high, from 71 to 94 as moderate, from 50 to 70 as low, and <50 as without support (Hillis and Bull, 1993). Posterior probabilities ≥0.95 were considered high and <0.95 as low (Alfaro et al., 2003).

### Principal component analysis

Principal component analysis (PCA) and plotting were implemented using FactoMineR in R (Lê et al., 2008) using 45 morphometric characters of protargol-impregnated specimens. The data were standardized by the function PCA in FactoMineR.

## Results

ZooBank Registration of the Present work: urn:lsid:zoobank.org:pub:C8448E69-D66B-412D-BCBD-F722A39D1396.

ZooBank Registration of *Tetmemena polymorpha* n. sp.: urn:lsid:zoobank.org:act:193CD898-1424-4477-B745-C25F0CF9D2C4.

### Taxonomy

Phylum Ciliophora Doflein, 1901

Subphylum Intramacronucleata Lynn, 1996

Class Spirotrichea Bütschli, 1889

Subclass Hypotrichia Stein, 1859

Family Oxytrichidae Ehrenberg, 1830

Subfamily Stylonychinae Berger and Foissner, 1997

Genus *Tetmemena* Eigner, 1999

**Figure 1:**
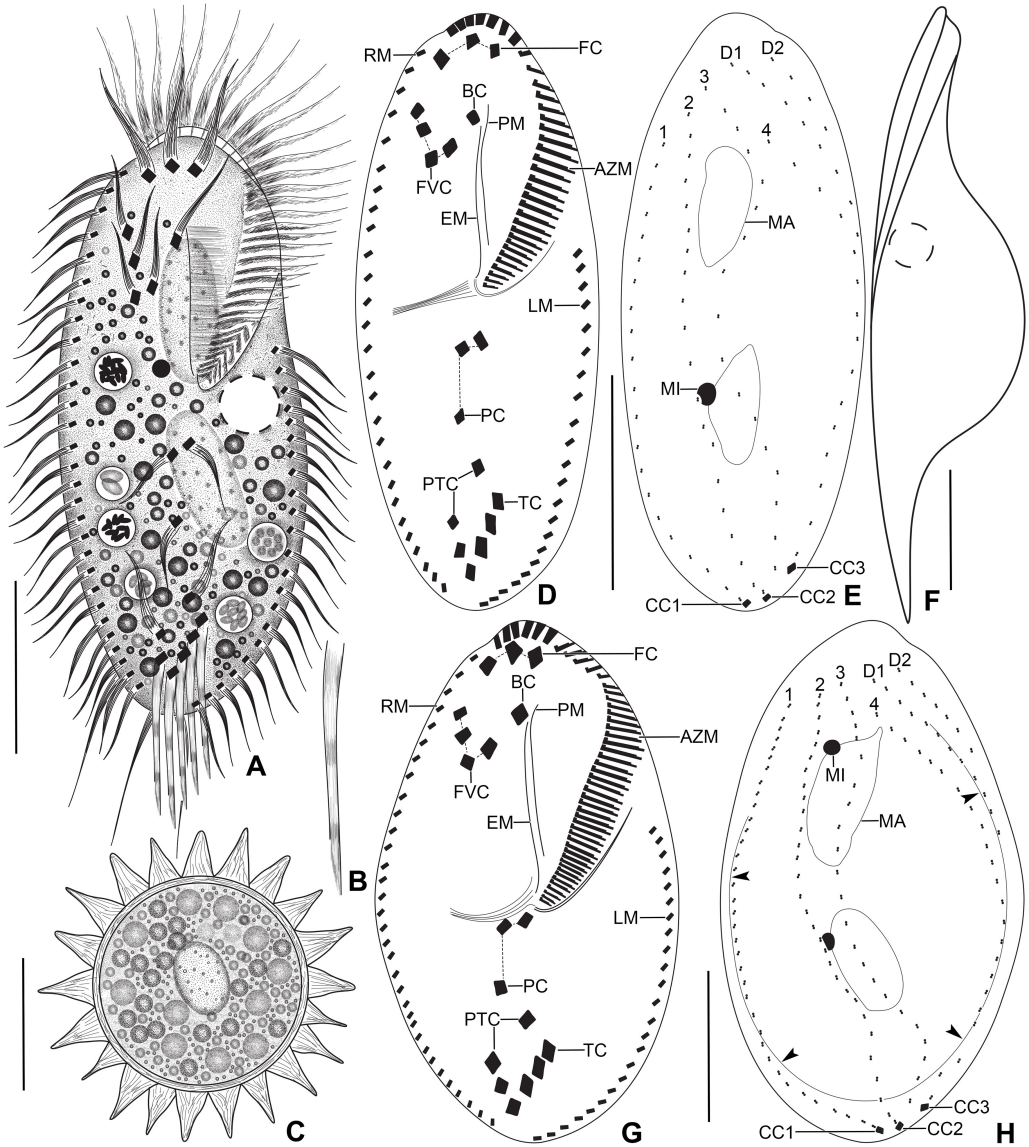
*Tetmemena polymorpha* n. sp. from life **(A–C, F)** and after protargol impregnation **(D, E, G, H)**. **(A)** Ventral view of a representative small morph specimen. **(B)** Transverse cirrus with the fringed posterior end. **(C)** Mature resting cyst, showing the thick wall, the large spines, and the single macronuclear nodule. **(D, E)** Ventral and dorsal view of a small morph hapantotype specimen, showing the infraciliature. **(F)** Left lateral view of a large morph specimen. **(G, H)** Ventral and dorsal view of a large morph hapantotype specimen, showing the broadly elliptical body, the large adoral zone of membranelles, and the distinctly bulged dorsal side (arrowheads). 1–4, dorsal kineties; AZM, adoral zone of membranelles; BC, buccal cirrus; CC1–3, caudal cirri; D1, 2, dorsomarginal kineties; EM, endoral membrane; FC, frontal cirri; FVC, frontoventral cirri; LM, left marginal row; MA, macronuclear nodule; MI, micronucleus; PC, postoral cirri; PM, paroral membrane; PTC, pretransverse cirri; RM, right marginal row; TC, transverse cirri. Scale bars 30 μm.

**Figure 2:**
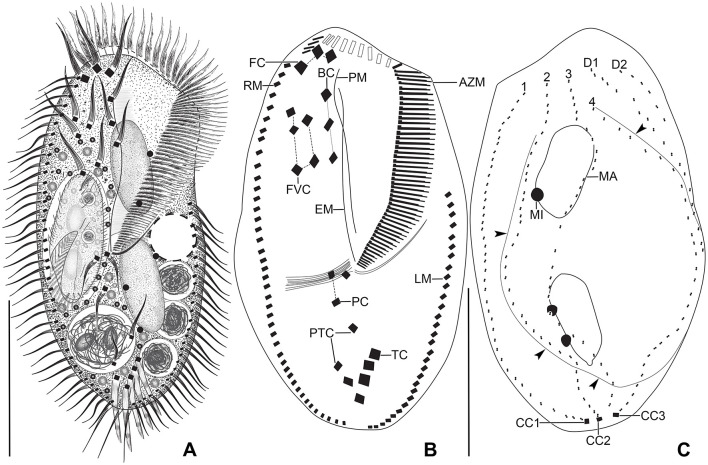
*Tetmemena polymorpha* n. sp., giant morph from life **(A)** and after protargol impregnation **(B, C)**. **(A)** Ventral view of a representative specimen, showing the body shape and the ventral cirral pattern. **(B, C)** Ventral and dorsal view of a hapantotype specimen, showing the large adoral zone with high number of membranelles, the anteriorly shortened dorsal kinety 4, and the distinctly bulged dorsal side (arrowheads). Dotted line connects buccal cirri, dashed lines connect frontoventral and postoral cirri, respectively. 1–4, dorsal kineties; AZM, adoral zone of membranelles; BC, buccal cirrus; CC1–3, caudal cirri; D1, 2, dorsomarginal kineties; EM, endoral membrane; FC, frontal cirri; FVC, frontoventral cirri; LM, left marginal row; MA, macronuclear nodule; MI, micronucleus; PC, postoral cirri; PM, paroral membrane; PTC, pretransverse cirri; RM, right marginal row; TC, transverse cirri. Scale bars 100 μm **(A)** and 50 μm **(B, C)**.

**Figure 3:**
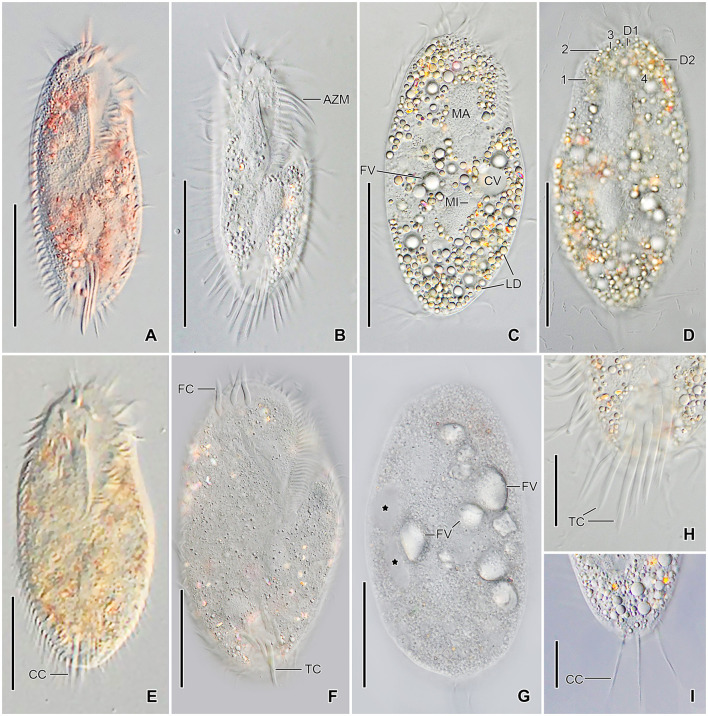
*Tetmemena polymorpha* n. sp., small **(A–D, H, I)** and large morph **(E–G)** specimens from life. **(A, B)** Ventral views showing the narrow body shape. **(C, D)** Ventral and dorsal view showing the cytoplasm studded with lipid droplets and the food vacuoles containing yeast cells. **(E–G)** Ventral **(E, F)** and dorsal **(G)** view, showing the broadly elliptical body, the food vacuoles containing starch grains. Asterisks mark the contractile vacuole and a collecting canal. **(H, I)** Ventral **(H)** and dorsal **(I)** view, showing the fringed transverse cirri and the long straight caudal cirri. 1–3, dorsal kineties; AZM, adoral zone of membranelles; CC, caudal cirri; CV, contractile vacuole; D1, 2, dorsomarginal kineties; FC, frontal cirri; FV, food vacuoles; LD, lipid droplets; MA, macronuclear nodule; TC, transverse cirri. Scale bars 50 μm **(A–G)** and 20 μm **(H, I)**.

**Figure 4:**
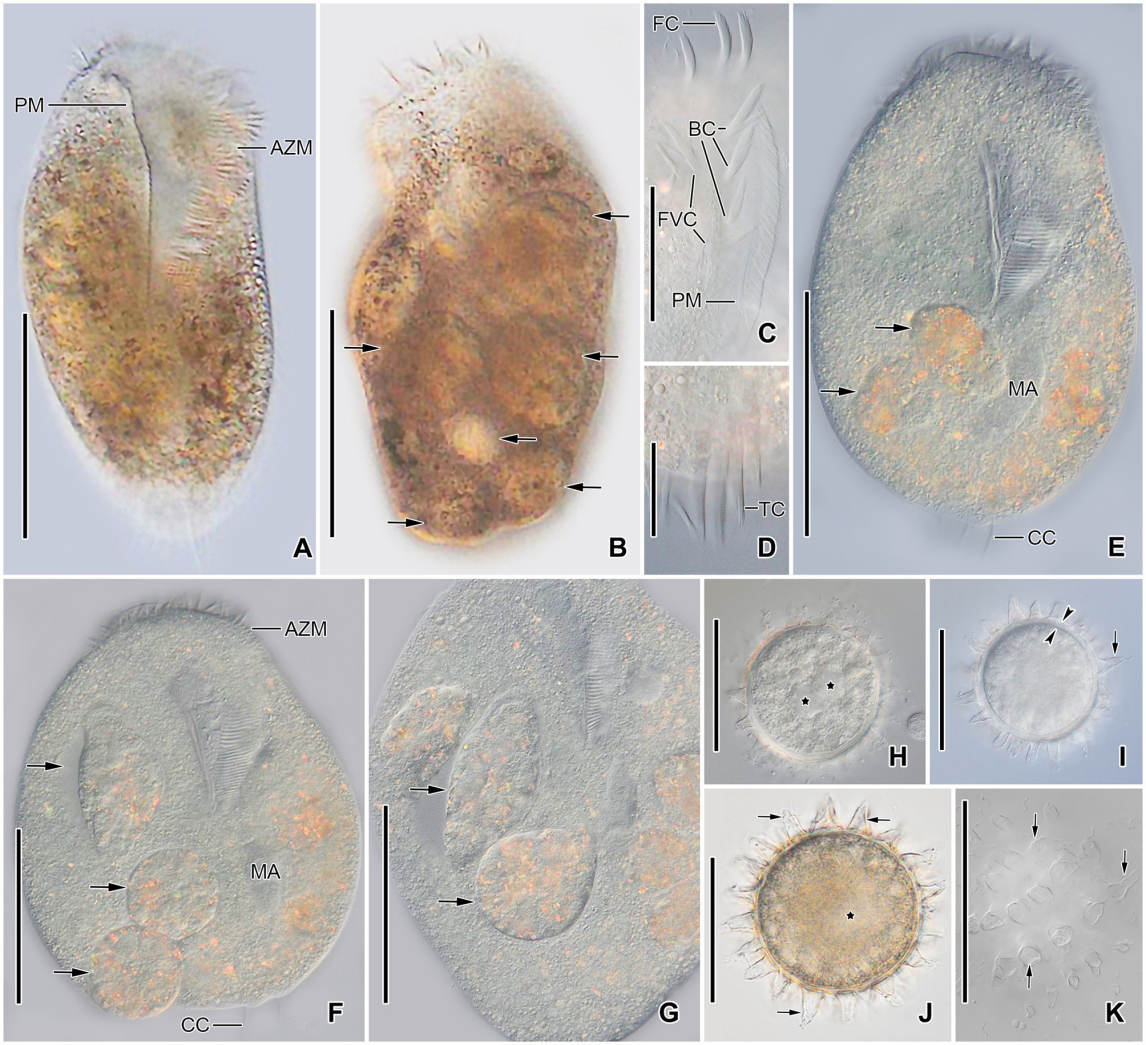
*Tetmemena polymorpha* n. sp., giant morph **(A–G)** specimens and resting cysts **(H–K)** from life. **(A, B)** Ventral **(A)** and dorsal **(B)** view of freely motile specimens, showing the body shape, the large adoral zone of membranelles, the very long paroral membrane, and the large food vacuoles (arrows). **(C)** Ventral view of the anterior portion of the body. **(D)** Ventral view of the posterior portion of body, showing the transverse cirri distinctly protruding from posterior body end. **(E–G)** Ventral views of slightly squashed cells, showing the nuclear apparatus and the food vacuoles containing small morph cells (arrows). **(H–K)** Optical sections **(H–J)** and surface view **(K)** of developing **(H)** and mature resting cysts **(I–K)**, showing the thick wall (opposite arrowheads), the large spines (arrows), and the macronuclear nodules (asterisks). Note the macronuclear nodules fuse after cyst maturation. AZM, adoral zone of membranelles; BC, buccal cirri; CC, caudal cirri; FC, frontal cirri; FVC, frontoventral cirri; MA, macronuclear nodules; PM, paroral membrane; TC, transverse cirri. Scale bars 100 μm **(A, B, E–G)**, 50 μm **(C, H–K)**, and 25 μm **(D)**.

**Figure 5:**
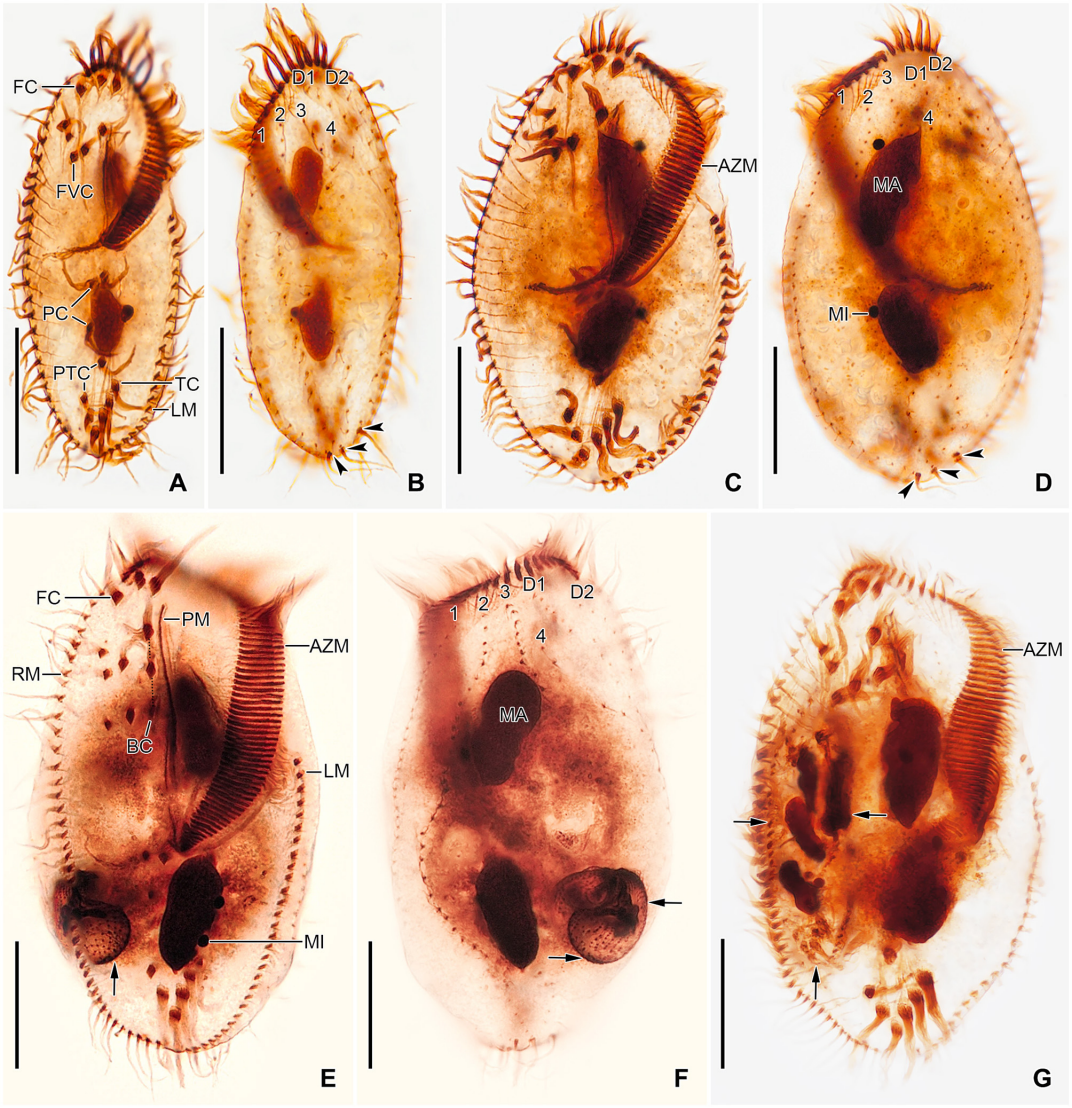
*Tetmemena polymorpha* n. sp., small **(A, B)**, large **(C, D)**, and giant morph **(E–G)** specimens after protargol impregnation. **(A, B)** Ventral and dorsal view of a hapantotype specimen, showing the narrowly elliptical body and the infraciliature. Arrowheads mark the caudal cirri. **(C, D)** Ventral and dorsal view of a hapantotype specimen, showing the broad body, the narrow anterior and posterior end, and the large adoral membranelles. Arrowheads show caudal cirri. **(E–G)** Ventral **(E, G)** and dorsal **(F)** view of a hapantotype **(E, F)** and a paratype **(G)** specimen, showing the truncated anterior end, the huge adoral zone, and the very long undulating membranes. Arrows mark the food vacuoles containing *Dexiostoma* sp. **(E, F)** and a small morph cell of *T. polymorpha* n. sp. **(G)**. 1–4, dorsal kineties; AZM, adoral zone of membranelles; BC, buccal cirrus; D1, 2, dorsomarginal kineties; FC, frontal cirri; FVC, frontoventral cirri; LM, left marginal row; MA, macronuclear nodule; MI, micronucleus; PC, postoral cirri; PM, paroral membrane; PTC, pretransverse cirri; RM, right marginal row; TC, transverse cirri. Scale bars 30 μm.

**Figure 6:**
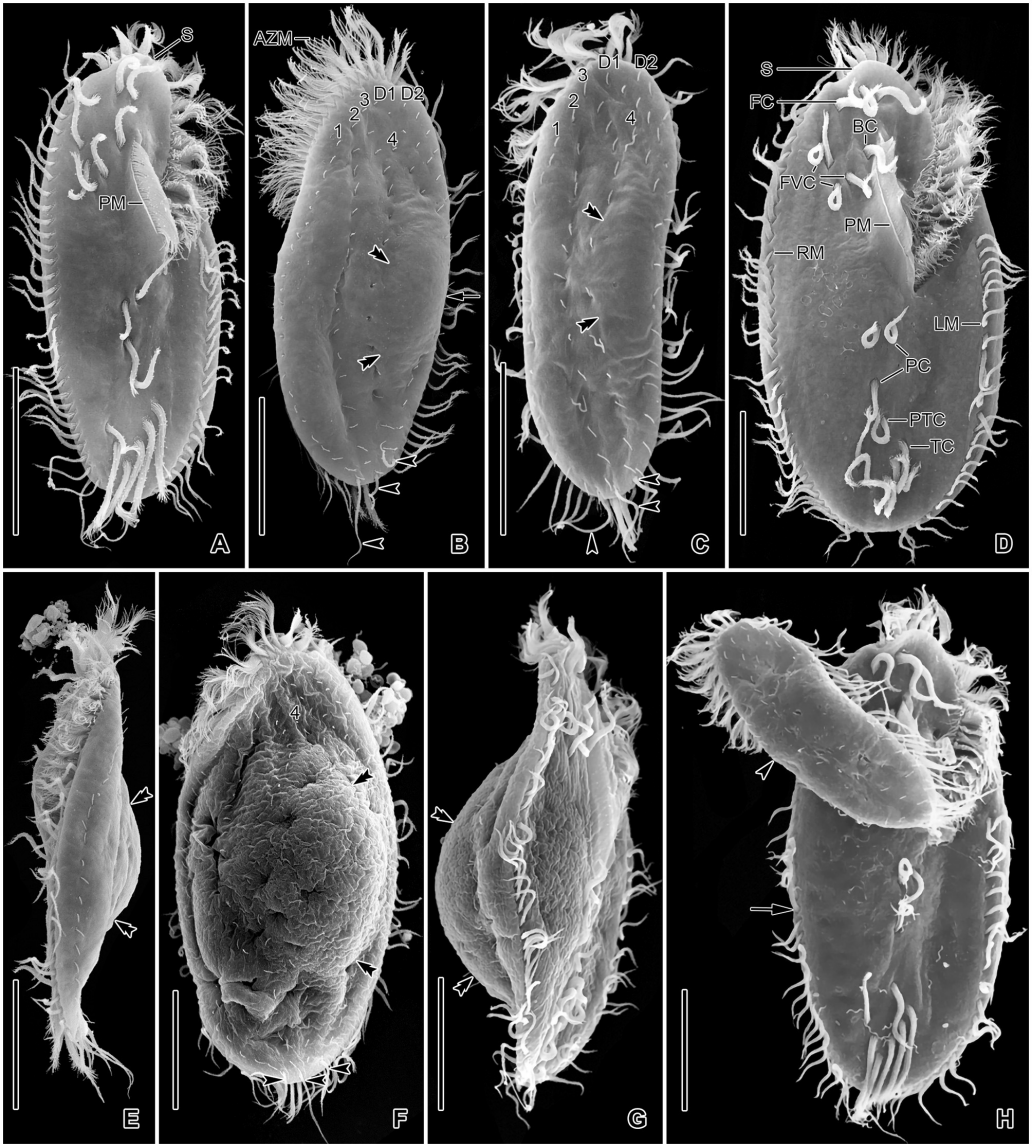
*Tetmemena polymorpha* n. sp. in the scanning electron microscope. **(A–C, E)** Ventral **(A)**, dorsal **(B, C)**, and left lateral **(E)** view of small morph specimens, showing the body shape, the ventral cirral pattern, the dorsal ciliature, the caudal cirri (arrowheads), the narrowly rounded scutum, and the indistinct dorsal bulge (double arrowheads). **(D, F, G)** Ventral **(D)**, dorsal **(F)**, and right lateral **(G)** view of large morph specimens showing the broad body, the narrowly rounded scutum, and the ventral cirral pattern, the caudal cirri (arrowheads) and the distinct dorsal bulge (double arrowheads). **(H)** A large morph (arrow) and a small morph (arrowhead) specimen showing different body sizes and shapes. 1–4, dorsal kineties; AZM, adoral zone of membranelles; BC, buccal cirrus; D1, 2, dorsomarginal kineties; FC, frontal cirri; FVC, frontoventral cirri; LM, left marginal row; PC, postoral cirri; PM, paroral membrane; PTC, pretransverse cirri; RM, right marginal row; S, scutum; TC, transverse cirri. Scale bars 30 μm.

**Figure 7:**
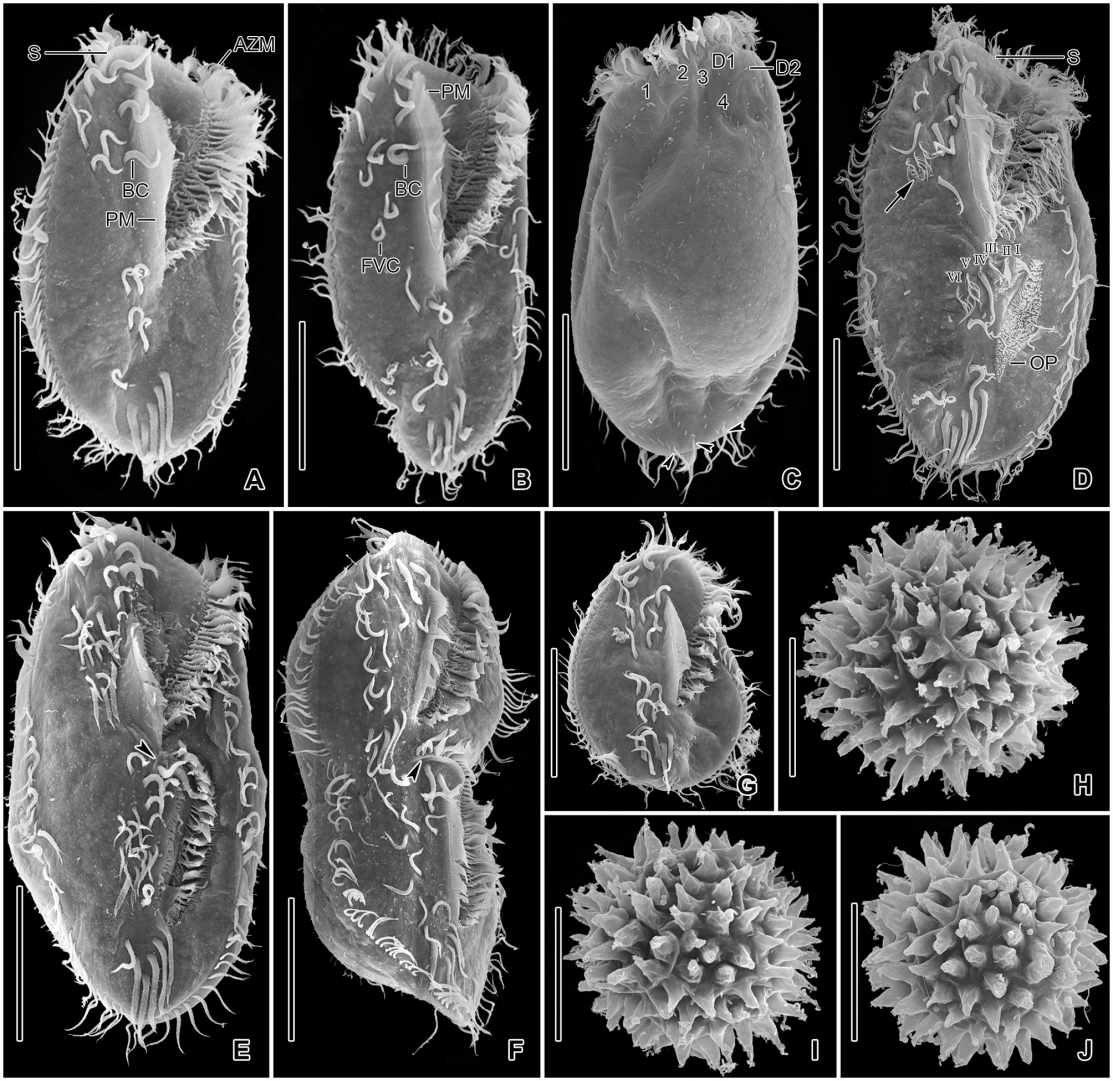
*Tetmemena polymorpha* n. sp., giant morph specimens **(A–G)** and resting cysts **(H–J)** in the scanning electron microscope. **(A–C)** Ventral **(A, B)** and dorsal **(C)** view, showing the body shape, the two buccal cirri, the four or five frontoventral cirri, the long paroral membrane, the broad and truncated scutum, the distinctly shortened dorsal kinety 4, and the three caudal cirri (arrowheads). **(D)** An early divider showing (i) the oral primordium forming anlagen I–III of the opisthe, (ii) the cirrus IV/3 dedifferentiates and forms three anlagen IV–VI of the proter (arrow), (iii) the cirrus V/4 dedifferentiate to form anlagen V and VI of the opisthe. **(E, F)** Late dividers, showing the ventral cirral pattern of the proter and opisthe. Arrowheads mark the scutum of the opisthe. **(G)** A post divider. **(H–J)** Surface views of mature resting cysts showing the spines. 1–4, dorsal kineties; AZM, adoral zone of membranelles; BC, buccal cirrus; D1, 2, dorsomarginal kineties; FVC, frontoventral cirri; I–VI, frontal-ventral-transverse anlagen; OP, oral primordium; PM, paroral membrane; S, scutum. Scale bars 50 μm **(A–G)** and 30 μm **(H–J)**.

***Tetmemena polymorpha* n. sp**. (Figures 1–7; Table 1)

**Table 1:** Morphometric data on *Tetmemena polymorpha* n. sp. small morph (first line), large morph (second line), and giant morph (third line).

**Characteristic^a^**	**H**	**Mean**	**M**	**SD**	**SE**	**CV**	**Min**	**Max**	***n***
Body, length (Ch1)	83.0	82.8	82.0	5.9	1.3	7.1	74.0	96.0	21
	104.0	110.7	111.0	5.9	1.3	5.3	100.0	123.0	21
	119.0	139.9	141.0	11.3	2.5	8.1	119.0	162.0	21
Body, width (Ch2)	34.0	36.9	35.0	5.3	1.2	14.4	30.5	52.0	21
	61.0	68.7	68.9	4.8	1.1	7.0	61.0	79.0	21
	69.0	84.3	83.0	11.4	2.5	13.5	63.0	103.0	21
Body length:width, ratio (Ch3)	2.4	2.3	2.3	0.2	0.1	9.0	1.7	2.6	21
	1.7	1.6	1.6	0.1	0.1	7.6	1.4	1.9	21
	1.7	1.7	1.7	0.2	0.1	9.5	1.4	1.9	21
Body, length *in vivo*	–	111.4	115.0	12.1	2.6	10.9	87.0	126	21
	–	151.1	151.0	10.2	2.0	6.8	136.0	170.0	27
	–	219.5	221.5	8.5	2.1	3.9	200.0	232.0	16
Body, width *in vivo*	–	42.5	42.0	6.0	1.3	14.0	33.0	53.0	21
	–	70.5	71.0	6.7	1.3	9.5	59.0	81.0	27
	–	109.6	111.0	9.0	2.2	8.2	90.0	132.0	16
Body length:width, ratio *in vivo*	–	2.6	2.6	0.3	0.1	12.3	1.7	3.1	21
	–	2.1	2.1	0.1	0.1	4.1	2.0	2.3	27
	–	2.0	2.0	0.1	0.1	6.4	1.7	2.3	16
Anterior body end to distal end of AZM, distance	4.0	4.7	4.7	0.6	0.1	13.6	3.9	5.9	21
	4.3	6.2	6.0	0.9	0.2	14.5	4.3	8.0	21
	7.9	7.1	7.0	1.4	0.3	19.9	5.0	10.0	21
Anterior body end to distal end of AZM, % of body length	4.8	5.7	5.5	0.7	0.2	12.4	4.8	7.2	21
	4.1	5.6	5.6	0.9	0.2	15.6	4.1	7.3	21
	6.6	5.1	4.8	1.3	0.3	25.9	3.4	8.0	21
Anterior body end to proximal end of AZM, distance (Ch4)	39.0	38.7	38.0	3.7	0.8	9.7	34.0	47.0	21
	58.0	57.3	58.0	3.0	0.6	5.2	51.0	63.0	21
	72.0	83.4	82.0	6.7	1.5	8.1	72.0	99.0	21
Body length:AZM, ratio	2.1	2.1	2.1	0.1	0.1	5.0	1.9	2.3	21
	1.8	1.9	1.9	0.1	0.1	5.1	1.8	2.2	21
	1.7	1.7	1.7	0.1	0.1	7.0	1.5	1.9	21
Adoral zone of membranelles, % of body length	47.0	46.6	46.9	2.4	0.5	5.0	42.7	51.4	21
	55.8	51.8	52.5	2.6	0.6	4.9	45.5	55.8	21
	60.5	59.1	58.3	4.2	0.9	7.1	51.4	67.8	21
DE-value (Ch5)	0.10	0.12	0.12	0.00	0.01	9.39	0.10	0.14	21
	0.07	0.11	0.11	0.02	0.01	14.88	0.07	0.14	21
	0.11	0.09	0.08	0.02	0.01	24.19	0.05	0.13	21
Adoral membranelles, number (Ch6)	34.0	33.4	33.0	3.0	0.6	8.9	28.0	41.0	21
	48.0	47.3	48.0	1.7	0.4	3.6	43.0	50.0	21
	63.0	67.9	67.0	5.1	1.1	7.5	62.0	83.0	21
Adoral membranelles, width of longest base (Ch8)	5.9	5.9	5.9	0.5	0.1	9.1	4.8	7.6	21
	8.6	8.4	8.3	0.6	0.1	7.2	7.4	10.0	21
	13.0	14.2	14.0	1.7	0.4	11.7	12.0	18.0	21
Gap between AZM and PM (maximum width of buccal cavity) (Ch7)^b^	7.6	7.1	6.7	1.2	0.3	16.9	5.6	10.0	21
	14.6	12.7	12.0	1.8	0.4	13.9	10.4	15.6	21
	19.0	23.3	23.0	4.8	1.0	20.5	13.0	31.0	21
Anterior body end to RMR, distance (Ch9)	5.3	5.7	5.7	0.7	0.2	12.9	4.0	7.4	21
	7.0	7.4	7.5	1.5	0.3	20.7	5.0	10.0	21
	9.0	9.5	10.0	2.0	0.4	21.0	6.7	14.0	21
Anterior body end to RMR, % of body length	6.4	6.9	7.0	0.9	0.2	12.4	5.1	8.3	21
	6.7	6.7	6.7	1.4	0.3	21.5	4.5	9.6	21
	7.6	6.9	6.8	1.7	0.4	24.9	4.5	11.2	21
Posterior body end to posterior end of RMR, distance	2.8	2.7	2.5	1.0	0.2	39.1	1.0	5.4	21
	2.7	2.3	2.5	1.5	0.3	66.8	0.0	6.3	21
	2.2	2.2	2.0	1.7	0.4	75.9	0.0	5.8	21
Right marginal row, number of cirri (Ch10)	27.0	29.5	29.0	2.2	0.5	7.3	26.0	33.0	21
	33.0	33.3	33.0	1.7	0.4	5.0	30.0	37.0	21
	42.0	38.2	38.0	3.1	0.7	8.1	31.0	44.0	21
Anterior body end to LMR, distance (Ch11)	32.0	32.1	32.0	2.5	0.6	7.9	28.0	38.0	21
	40.0	40.6	41.4	3.0	0.7	7.4	35.0	45.0	21
	52.0	57.0	55.0	7.9	1.7	13.8	40.0	70.0	21
Anterior body end to LMR, % of body length	38.6	38.7	38.6	1.5	0.3	3.8	35.0	40.9	21
	38.5	36.7	36.9	2.4	0.5	6.5	31.8	39.7	21
	43.7	40.7	40.3	4.8	1.0	11.7	32.0	50.8	21
Posterior body end to posterior end of LMR, distance	0.5	0.7	0.7	0.5	0.1	–	0.0	1.8	21
	0.5	0.4	0.0	0.5	0.1	–	0.0	1.2	21
	0.0	0.0	0.0	0.2	0.1	–	0.0	1.0	21
Left marginal row, number of cirri (Ch12)	22.0	21.7	22.0	1.4	0.3	6.4	19.0	24.0	21
	24.0	23.7	24.0	1.8	0.4	7.5	20.0	26.0	21
	30.0	28.2	28.0	3.0	0.7	10.8	22.0	33.0	21
Anterior body end to BC, distance (Ch13)	14.0	13.6	13.5	0.9	0.2	6.6	12.0	16.0	21
	16.0	16.8	16.0	1.8	0.4	10.6	14.9	21.0	21
	17.0	17.3	17.0	2.3	0.5	13.6	13.0	22.0	21
Anterior body end to BC, % of body length	16.9	16.5	16.7	0.9	0.2	5.4	15.1	18	21
	15.4	15.1	15.3	1.1	0.3	7.6	13.4	17.1	21
	14.3	12.4	12.6	1.7	0.4	13.5	9.8	14.8	21
Anterior body end to PM, distance (Ch14)	13.7	13.5	13.6	1.0	0.2	7.3	11.6	16.0	21
	16.0	16.3	17.0	1.4	0.3	8.8	13.0	18.0	21
	13.0	12.1	12.5	2.6	0.6	21.0	7.0	19.0	21
Anterior body end to PM, % of body length	16.5	16.4	16.6	0.9	0.2	5.6	13.6	18.2	21
	15.4	14.7	14.7	1.3	0.3	9.1	11.6	16.7	21
	10.9	8.8	8.4	2.0	0.4	23.0	5.0	13.0	21
Paroral membrane, length (Ch15)	18.7	17.9	18.0	2.7	0.6	14.9	13.0	24.0	21
	28.0	29.6	29.0	2.5	0.5	8.4	25.0	34.9	21
	47.0	56.8	57.0	5.2	1.1	9.1	45.0	64.0	21
Anterior body end to EM, distance (Ch16)	17.5	17.0	17.0	1.1	0.2	6.4	15.7	20.0	21
	19.6	20.4	20.8	1.3	0.3	6.4	18.0	23.0	21
	17.0	17.6	17.7	2.3	0.5	13.2	12.0	23.0	21
Anterior body end to EM, % of body length	21.1	20.6	20.6	0.9	0.2	4.3	18.7	22.0	21
	18.8	18.4	18.4	1.1	0.2	5.9	16.1	20.4	21
	14.3	12.6	12.8	1.8	0.4	14.2	8.5	16.0	21
Endoral membrane, length (Ch17)	20.0	19.3	19.0	2.8	0.6	14.5	14.0	26.0	21
	33.0	33.2	33.0	2.1	0.5	6.3	29.0	36.0	21
	50.0	60.6	61.0	5.8	1.3	9.5	50.0	72.0	21
Anterior body end to anterior macronuclear nodule, distance (Ch18)	19.0	18.6	18.0	1.9	0.4	10.4	16.0	23.0	21
	21.6	26.1	24.9	3.3	0.7	12.8	21.6	35.0	21
	29.0	35.3	36.0	3.8	0.8	10.8	28.0	42.0	21
Posterior body end to posterior macronuclear nodule, distance	20.0	20.6	20.0	1.9	0.4	9.4	16.0	24.5	21
	25.5	27.5	27.0	3.1	0.7	11.1	23.0	35.0	21
	19.0	29.3	29.0	5.4	1.2	18.3	19.0	40.0	21
Anterior macronuclear nodule, length (Ch19)	16.6	16.3	16.6	1.9	0.4	11.4	13.0	20.0	21
	30.0	26.4	26.0	2.5	0.6	9.6	23.0	31.0	21
	28.0	31.9	33.0	4.3	0.9	13.4	21.0	41.0	21
Anterior macronuclear nodule, width (Ch20)	6.5	7.1	7.0	0.7	0.2	9.8	5.8	8.0	21
	12.0	12.4	12.4	1.2	0.3	9.4	10.3	14.0	21
	13.0	13.5	13.0	1.4	0.3	10.6	12.0	17.0	21
Macronuclear nodules, number (Ch21)	2.0	2.0	2.0	0.0	0.0	0.0	2.0	2.0	21
	2.0	2.0	2.0	0.0	0.0	0.0	2.0	2.0	21
	2.0	2.0	2.0	0.0	0.0	0.0	2.0	2.0	21
Micronuclei, diameter	2.4	2.6	2.6	0.3	0.1	12.4	2.0	3.3	21
	2.8	3.0	3.0	0.3	0.1	10.4	2.4	4.0	21
	3.3	3.0	3.0	0.1	0.1	2.2	3.0	3.3	21
Micronuclei, number (Ch22)	1.0	1.4	1.0	0.6	0.1	41.8	1.0	3.0	21
	2.0	2.0	2.0	0.6	0.1	31.6	1.0	3.0	21
	3.0	4.5	4.0	1.6	0.3	35.8	1.0	8.0	21
Nuclear figure, length (Ch23)	44.0	44.4	44.0	4.0	0.9	9.0	38.0	54.0	21
	58.0	58.7	58	4.7	1.0	7.9	51.3	68.0	21
	70.0	75.4	75.0	7.2	1.6	9.6	60.0	87.0	21
Macronuclear nodules, distance in between	11.0	10.9	11.3	2.0	0.4	18.2	6.3	13.5	21
	9.4	10.0	9.4	3.9	0.9	39.3	3.0	16.0	21
	17.0	9.9	10.0	8.7	1.9	88.1	0.0	28.0	21
Frontal cirri, number (Ch24)	3.0	3.0	3.0	0.0	0.0	0.0	3.0	3.0	21
	3.0	3.0	3.0	0.0	0.0	0.0	3.0	3.0	21
	3.0	3.0	3.0	0.0	0.0	0.0	3.0	3.0	21
Buccal cirri, number (Ch25)	1.0	1.0	1.0	0.0	0.0	0.0	1.0	1.0	21
	1.0	1.1	1.0	0.4	0.1	31.4	1.0	2.0	21
	3.0	2.8	3.0	0.4	0.1	15.8	2.0	3.0	21
Anterior body end to anterior frontoventral cirrus, distance	14.0	13.3	13.5	1.3	0.3	9.6	11.0	16.0	21
	17.5	17.5	17.6	1.9	0.4	10.6	14.0	21.0	21
	23.0	22.8	23.0	3.8	0.8	16.8	15.0	29.0	21
Anterior body end to end of frontoventral cirri, distance	23.0	22.1	22.0	1.3	0.3	5.9	20.3	25.0	21
	30.0	30.7	30.4	2.4	0.5	7.8	25.0	37.0	21
	43.0	46.5	46.0	7.4	1.6	16.0	34.0	61.0	21
Frontoventral cirri, number (Ch26)	4.0	4.0	4.0	0.0	0.0	0.0	4.0	4.0	21
	4.0	4.0	4.0	0.0	0.0	0.0	4.0	4.0	21
	5.0	4.8	5.0	0.5	0.1	11.3	4.0	6.0	21
Anterior body end to anterior postoral cirrus, distance	44.0	41.9	41.0	3.6	0.8	8.6	36.0	50.0	21
	58.0	59.1	58.8	3.2	0.7	5.4	54.0	65.0	21
	71.0	82.7	82.0	7.5	1.6	9.0	71.0	99.0	21
Anterior body end to posterior postoral cirrus, distance	55.0	52.2	51.0	4.0	0.9	7.7	45.7	62.0	21
	71.0	73.4	72.0	4.0	0.9	5.4	66.0	81.0	21
	80.0	97.0	96.0	8.5	1.9	8.8	80.0	112.0	21
Postoral cirri, number (Ch27)	3.0	3.0	3.0	0.0	0.0	0.0	3.0	3.0	21
	3.0	3.0	3.0	0.0	0.0	0.0	3.0	3.0	21
	3.0	3.0	3.0	0.2	0.1	7.2	3.0	4.0	21
Pretransverse cirri, number (Ch28)	2.0	2.0	2.0	0.0	0.0	0.0	2.0	2.0	21
	2.0	2.0	2.0	0.0	0.0	0.0	2.0	2.0	21
	2.0	2.0	2.0	0.0	0.0	0.0	2.0	2.0	21
Posterior body end to anterior pretransverse cirrus, distance	18.7	17.9	18.0	2.2	0.5	12.4	14.0	22.6	21
	22.8	24.9	25.0	1.8	0.4	7.1	22.0	28.0	21
	29.0	29.4	29.0	3.8	0.8	12.8	23.0	36.0	21
Posterior body end to posterior pretransverse cirrus, distance	11.3	10.6	10.5	1.5	0.3	13.8	7.7	13.5	21
	13.7	15.4	15.0	1.7	0.4	11.2	12.3	19.0	21
	18.0	18.4	19.0	3.0	0.7	16.5	12.0	23.0	21
Transverse cirri, number (Ch29)	5.0	5.0	5.0	0.0	0.0	0.0	5.0	5.0	21
	5.0	5.0	5.0	0.0	0.0	0.0	5.0	5.0	21
	5.0	5.0	5.0	0.2	0.1	4.3	5.0	6.0	21
Posterior body end to posterior transverse cirrus, distance	4.3	3.8	3.5	0.8	0.2	21.8	2.6	6.2	21
	5.2	5.8	6.0	1.2	0.3	20.1	4.0	9.0	21
	7.5	7.1	7.5	2.0	0.4	28.6	2.0	10.0	21
Frontal-ventral-transverse cirri, total number (Ch30)	18.0	18.0	18.0	0.0	0.0	0.0	18.0	18.0	21
	18.0	18.1	18.0	0.4	0.1	2.0	18.0	19.0	21
	21.0	20.6	21.0	0.8	0.2	3.9	19.0	22.0	21
Dorsal kineties, number (Ch31)	6.0	6.0	6.0	0.0	0.0	0.0	6.0	6.0	21
	6.0	6.0	6.0	0.0	0.0	0.0	6.0	6.0	21
	6.0	6.2	6.0	0.4	0.1	6.5	6.0	7.0	21
Anterior body end to anterior end of DK1, distance (Ch32)	18.5	18.2	18.0	1.7	0.4	9.5	15.0	21.0	21
	16.0	18.8	19.0	2.4	0.5	12.6	12.7	22.4	21
	17.0	21.6	21.0	3.2	0.7	14.9	17.0	28.0	21
Dorsal kinety 1, number of bristles (Ch33)	21.0	21.5	21.0	1.9	0.4	8.6	18.0	27.0	21
	36.0	35.2	35.0	2.4	0.5	6.9	31.0	42.0	21
	42.0	45.9	46.0	3.8	0.8	8.3	38.0	52.0	21
Anterior body end to anterior end of DK2, distance (34)	14.3	14.4	14.0	1.1	0.2	7.5	12.6	17.0	21
	13.8	15.6	16.0	2.3	0.5	14.8	11.0	19.3	21
	16.0	16.8	16.0	2.6	0.6	15.7	13.0	21.0	21
Dorsal kinety 2, number of bristles (Ch35)	20.0	19.6	20.0	1.6	0.4	8.3	16.0	24.0	21
	30.0	29.1	29.0	1.8	0.4	6.1	26.0	34.0	21
	38.0	37.1	38.0	3.5	0.8	9.4	29.0	42.0	21
Anterior body end to anterior end of DK3, distance (Ch36)	10.9	11.1	11.0	1.1	0.2	9.9	9.0	13.2	21
	11.4	13.2	13.0	1.2	0.3	8.9	11.0	15.0	21
	14.0	14.6	14.0	2.8	0.6	19.1	11.0	21.0	21
Dorsal kinety 3, number of bristles (Ch37)	17.0	16.8	17.0	1.3	0.3	7.7	15.0	20.0	21
	23.0	24.0	24.0	1.7	0.4	6.9	21.0	27.0	21
	32.0	32.3	33.0	3.2	0.7	10.0	27.0	39.0	21
Anterior body end to anterior end of DK4, distance (Ch38)	18.0	17.1	17.0	1.9	0.4	10.9	14.0	20.0	21
	17.9	20.0	19.7	2.9	0.6	14.6	15.0	27.0	21
	23.0	21.9	22.0	4.3	0.9	19.5	14.0	31.0	21
Dorsal kinety 4, number of bristles (Ch39)	15.0	15.0	15.0	1.3	0.3	8.8	12.0	17.0	21
	23.0	25.3	26.0	2.4	0.5	9.5	21.0	27.0	21
	29.0	30.1	30.0	3.4	0.7	11.1	24.0	35.0	21
Anterior body end to anterior end of DM1, distance (Ch40)	7.6	7.8	8.0	0.8	0.2	10.1	6.5	9.6	21
	11.0	11.4	11.0	1.6	0.3	13.6	9.0	15.0	21
	11.0	12.7	12.0	2.5	0.5	19.4	10.0	18.0	21
Dorsomarginal row 1, number of bristles (Ch41)	9.0	8.9	9.0	1.4	0.3	16.2	6.0	11.0	21
	12.0	13.6	13.0	1.6	0.4	12.0	10.0	17.0	21
	19.0	17.3	18.0	2.7	0.6	15.7	11.0	21.0	21
Anterior body end to anterior end of DM2, distance (Ch42)	7.6	7.7	7.6	0.8	0.2	11.0	6.0	9.9	21
	10.0	10.2	9.9	1.5	0.3	14.5	8.0	14.0	21
	14.0	11.7	11.0	1.6	0.3	13.5	10.0	15.0	21
Dorsomarginal row 2, number of bristles (Ch43)	4.0	3.4	3.0	0.7	0.1	19.7	2.0	5.0	21
	7.0	6.7	7.0	1.1	0.2	16.4	5.0	9.0	21
	6.0	8.0	8.0	1.8	0.4	22.0	5.0	12.0	21
Dorsal bristles, total number (Ch44)	86.0	85.2	85.0	5.7	1.2	6.7	75.0	103.0	21
	131.0	134.0	135.0	6.8	1.5	5.1	121.0	149.0	21
	167.0	170.8	171.0	11.3	2.5	6.6	146.0	187.0	21
Caudal cirri, number (Ch45)	3.0	3.0	3.0	0.0	0.0	0.0	3.0	3.0	21
	3.0	3.0	3.0	0.0	0.0	0.0	3.0	3.0	21
	3.0	3.0	3.0	0.0	0.0	0.0	3.0	3.0	21
Caudal cirrus 1 to caudal cirrus 2, distance	1.8	2.2	2.1	0.4	0.1	17.0	1.8	3.0	21
	2.5	3.1	3.0	0.5	0.1	15.2	2.4	3.9	21
	2.6	3.6	3.6	0.7	0.2	20.9	2.0	5.1	21
Caudal cirrus 2 to caudal cirrus 3, distance	4.1	4.2	4.1	0.5	0.1	11.0	3.4	5.1	21
	4.3	4.8	4.7	0.6	0.1	11.7	3.7	5.9	21
	3.5	5.3	5.2	1.0	0.2	18.1	3.5	7.2	21
Resting cysts, diameter *invivo*^c^	-	71.3	73.0	5.6	1.5	7.9	60.0	79.0	15
Resting cysts, diameter in SEM^c^	-	51.6	51.0	5.5	0.9	10.6	40.0	63.0	38

a Data based, if not mentioned otherwise, on protargol-impregnated specimens. Measurements in μm. Numbers in parentheses (CH1–45) indicate characters used in the principal component analysis. AZM, adoral zone of membranelles; BC, buccal cirrus; CV, coefficient of variation in %; DK, dorsal kinety; DM, dorsomarginal kinety; EM, endoral membrane; H, hapantotypes; LMR, left marginal row; M, median; Max, maximum; Mean, arithmetic mean; Min, minimum; n, number of individuals investigated; PM, paroral membrane; RMR, right marginal row; SD, standard deviation; SE, standard error of arithmetic mean; SEM, scanning electron micrographs.

b The transverse distance between the anterior end of the paroral membrane to the opposite adoral membranelle on the left side.

c Including spines.

### Diagnosis

Size of small morph (SM) *in vivo* 87–126 × 33–53 μm, that of large morph (LM) 136–170 × 59–81 μm, and that of cannibalistic giant morph (GM) 200–232 × 90–132 μm. Body of SM narrowly elliptical with anterior and posterior end slightly narrowed; LM broadly elliptical, anterior and posterior end narrowed, right and left side convex, dorsal side distinctly bulged; and GM broadly ellipsoidal with broad and distinctly truncated to left anterior end and narrowly rounded posterior end, hump of dorsal side irregular depending on feeding status. SM and LM with 18 frontal-ventral-transverse cirri, of which one buccal and four frontoventral; GM with 19–22 frontal-ventral-transverse cirri, of which two or three buccal and 4–6 frontoventral. Right marginal row composed of 26–33 cirri in SM, 30–37 cirri in LM, and 31–44 cirri in GM; left row composed of 19–24 cirri in SM, 20–26 cirri in LM, and 22–33 cirri in GM. Dorsal kinety 4 shortened anteriorly. Total number of dorsal dikinetids 75–103 in SM, 121–149 in LM, and 146–187 in GM. Three caudal cirri slightly to distinctly shifted to right, distance between cirri 1 and 2 narrower than distance between cirri 2 and 3. Adoral zone occupies 43–51% of body length and composed of 28–41 membranelles in SM, 46–56% of body length and composed of 43–50 membranelles in LM, and 51–68% of body length and composed of 62–83 membranelles in GM. Resting cysts 60–79 μm *in vivo* with spines 5–12 μm wide at bases and 10–15 μm long and fused macronuclear nodule.

### Etymology

The species-group name “*polymorpha*” is a composite of the Greek quantifier *polys* (many) and the Greek substantive *morphe* (shape), referring to the different morphs of *Tetmemena polymorpha* n. sp.

### Type locality

Estuarine area (salinity of 4.7‰) of Namdaecheon Stream in Gangneung-si, South Korea (37° 46′ 11″N, 128° 56′ 58″E).

### Type material

Three hapantotype slides with protargol-impregnated small, large, and giant morph specimens (NNIBRPR25452–NNIBRPR25454, respectively) have been deposited in the Nakdonggang National Institute of Biological Resources (NNIBR). Hapantotypes, paratypes, and other relevant specimens have been marked by black ink circles on the back of the slides. Nine slides (GUC006316–6318, GUC006320–6322, GUC006324, GUC006326, and GUC006327) have been deposited in the Jung-lab (J.-H. Jung) in Gangneung-Wonju National University.

### Morphological description of *Tetmemena polymorpha* n. sp.

Size of SM *in vivo* 87–126 × 33–53 μm (on average 111 × 43 μm); LM 136–170 × 59–81 μm (on average 151 × 71 μm); and GM 200–232 × 90–132 μm (on average 220 × 110 μm). Body of SM slim with anterior and posterior end slightly narrowed, right side usually slightly convex, rarely S-shaped, left side S-shaped, dorsal side with indistinct bulge, length:width ratio 1.7–3.1:1 *in vivo* (Figures 1A, D, E, 3A–D, 6A–C, E, H). Body of LM broadly elliptical, anterior and posterior end narrowed, right and left side convex, dorsal side distinctly bulged in central two thirds of cell, length:width ratio 2.0–2.3:1 *in vivo* (Figures 1F–H, 3E–G, 6D, F–H). Body of GM broadly elliptical with anterior end broad and distinctly truncated to the left and posterior end narrowly rounded, right side convex sometimes with a distinct concavity at the level of transverse cirri, left side straight or convex, hump of the dorsal side depends on feeding status from a central hump on middle quarters to irregular hump covering almost all dorsal surface, length:width ratio 1.7–2.3:1 *in vivo* (Figures 2A–C, 4A, B, 7A–C). Nuclear apparatus commences at about 22% of body length in SM and 25% of body length in both LM and GM, and ends at about 75% of body length in both SM and LM and about 80% of body length in GM. Invariably two macronuclear nodules in all morphs, 1–3 micronuclei in both SM and LM and 1–8 micronuclei in GM. Individual macronuclear nodules ellipsoidal to narrowly ellipsoidal, in or left to body's midline, *in vivo* 20–30 × 10–15 μm in SM, 25–40 × 10–20 μm in LM, and 40–60 × 15–25 μm in GM. After protargol impregnation, macronuclear nodules of GM are sometimes connected with thread-like structures. Micronuclei near or attached to macronuclear nodules, spherical, 3–5 μm across in all morphs (Figures 1A, E, H, 2C, 3C, 4E–G, 5A, D–F). Contractile vacuole in mid-body at left cell margin; at end of diastole, 8–10 μm across in SM, 12–15 μm in LM, and 23–30 μm in GM. Collecting canals present but hardly recognizable in most cells. Cortex rigid and colorless; cortical granules lacking (Figures 1A, 2A, 3C, G). Cytoplasm hyaline, studded with lipid droplets, refractive crystals, and food vacuoles up to 7 μm across containing only bacteria and single yeast cells in SM; up to 30 μm across containing yeast cells, starch grains, or small ciliate cells used as food (*Dexiostoma* sp. or *Tetrahymena* sp.) in LM; in GM, cytoplasm contains up to 11 food vacuoles, each contains a single SM cell up to 100 × 50 μm in size or small ciliate cells (Figures 3C, E, G, 4A, B, E–G, 5E–G).

Cirral pattern of *Tetmemena polymorpha* n. sp. usually in *Oxytricha* pattern, i.e., 18 frontal-ventral-transverse cirri, with few extra cirri in LM and GM. Invariably three enlarged frontal cirri, with cilia about 20 μm *in vivo* in both SM and LM and up to 30 μm long in GM. One buccal cirrus with the same length but with a narrower base than those of frontal cirri in SM; one (rarely two) buccal cirrus of the same size as frontal cirri in LM; two or three buccal cirri arranged in line right to the paroral membrane, 20–25 μm long *in vivo* in GM. Four frontoventral cirri arranged in the V-shaped pattern with cilia about 17 μm long in SM and about 20 μm long in LM *in vivo*; 4–6 frontoventral cirri with 20–25 μm long cilia *in vivo* arranged in two slightly oblique rows in GM. Invariably three postoral cirri in the inverted L-shape pattern, with the same length as frontoventral cirri, cirrus IV/2 placed more anteriorly than cirrus V/4 in both SM and LM while GM sometimes at the same level or even placed only slightly posteriorly. Two obliquely arranged pretransverse cirri, with the same length as frontoventral cirri. Invariably five transverse cirri arranged in the hook-shaped pattern, all cirri distinctly protrude beyond the posterior cell margin *in vivo*, fringed distally, about 25 μm long in both SM and LM and 30–35 μm long in GM (Figures 1A, B, D, E, G, H, 2A–C, 3A, B, E, F, H, 4C, D, 5A–G, 6A, D; Table 1). Marginal cirri fine, gradually slightly decreasing in size posteriorly, i.e., anterior cirrus about 20 μm long while posterior cirrus about 15 μm long *in vivo*. Right marginal row commences subapically at about 7% of body length and ends terminally, composed of 26–33 cirri in SM, 30–37 cirri in LM, and 31–44 cirri in GM. Left marginal row commences in the second quarter of the cell and extends to the body's midline posteriorly, composed of 19–24 cirri in SM, 20–26 cirri in LM, and 22–33 cirri in GM. The gap between posterior ends of marginal rows slightly shifter to right in both SM and LM while usually in the midline in GM (Figures 1A, D, E, G, H, 2A–C, 5A, C, E, 6A, D, 7A–C, G).

Six dorsal kineties, including two dorsomarginal ones, with bristles 3–4 μm long *in vivo* in all morphs: kineties 1–3 bipolar, not curved anteriorly; kinety 4 distinctly shorter than kineties 1–3 anteriorly. Dorsomarginal kineties 1 and 2 extend posteriorly over one-third and one-fifth of cell length, respectively, in both SM and LM; in GM, dorsomarginal kineties 1 and 2 extend posteriorly to mid-body and first quarter of cell, respectively. The number of dorsal bristles significantly differs among morphs, i.e., on average a total of 85 dikinetids in SM, 134 dikinetids in LM, and 171 dikinetids in GM. Three straight caudal cirri, about 25 μm long *in vivo* in SM and LM, and 30–35 μm long in GM, slightly shifted to right, at the ends of dorsal kineties 1, 2, and 4; distance between cirri 1 and 2 narrower than the distance between cirri 2 and 3; cirrus 3 optically at the level between second and third-last cirri of right marginal row (Figures 1E, H, 2C, 3D, I, 4E, F, 5B, D, F, 6B, C, F, H, 7C; Table 1).

The adoral zone occupies about 47% of body length and is composed of about 34 membranelles in SM, about 52% of body length and composed of about 47 membranelles in LM, and about 59% of body length and is composed of about 68 membranelles in GM. The distal end of the adoral zone commences at averages of 5.7, 5.6, and 5.1% of body length on the right side (DE-values 0.12, 0.11, and 0.09 on average) in SM, LM, and GM, respectively. Cilia of membranelles 20–25 μm long in both SM and LM and up to 30 μm in GM *in vivo*, bases of largest membranelles about 6 μm wide in SM, 8 μm wide in LM, and 14 μm wide in GM after protargol impregnation. Frontal scutum narrowly rounded in both SM and LM, wide and truncated to left in GM. Buccal cavity narrow (6–10 μm wide) in SM to relatively narrow (10–16 μm wide) in LM anteriorly and comparatively wide in GM (13–31 μm wide in protargol preparations) and narrowing posteriorly (Figures 1A, D, G, 2A, B, 3A, B, E, F, 4A, E, 5A, C, E, G, 6A, D, 7A, B, D). Undulating membranes in *Stylonychia* pattern, i.e., parallel or slightly overlapping: paroral commences anterior to buccal cirrus at about 16.4% of body length with a length of about 18 μm in SM; begins at about 14.7% of body length with a length of about 30 μm in LM; and begins more anteriorly, at about 8.8% of body length, and with a length of about 57 μm in protargol-impregnated specimens and extends to near the end of buccal vertex in GM. Endoral slightly longer than paroral, commences posterior to anterior end of paroral and extends to the end of the buccal vertex. Membranes at body's midline, cilia about 8 μm long in SM, about 10 μm long in LM and about 15 μm long in GM (Figures 1A, D, G, 2A, B, 4A, C, 5A, C, E, 6A, D, 7A, B, D). Pharyngeal fibers extend transversely to the right body margin (Figures 1D, G, 2B, 5A, C, E).

### Resting cysts

Cysts of *Tetmemena polymorpha* n. sp. spherical, size including spines 60–79 μm (on average 71 μm) across *in vivo* and 40–63 μm (on average 52 μm) across in SEM preparations. Wall hyaline about 3–4 μm thick *in vivo*, ornamented by thick spines, 5–12 μm wide at the base and 10–15 μm long each. Cytoplasm studded with lipid droplets; macronuclear nodules separate in developing cysts and fuse to form a single macronuclear nodule in mature cysts (Figures 1C, 4H–K, 7H–J; Table 1).

### Notes on ontogenesis

**Figure 8:**
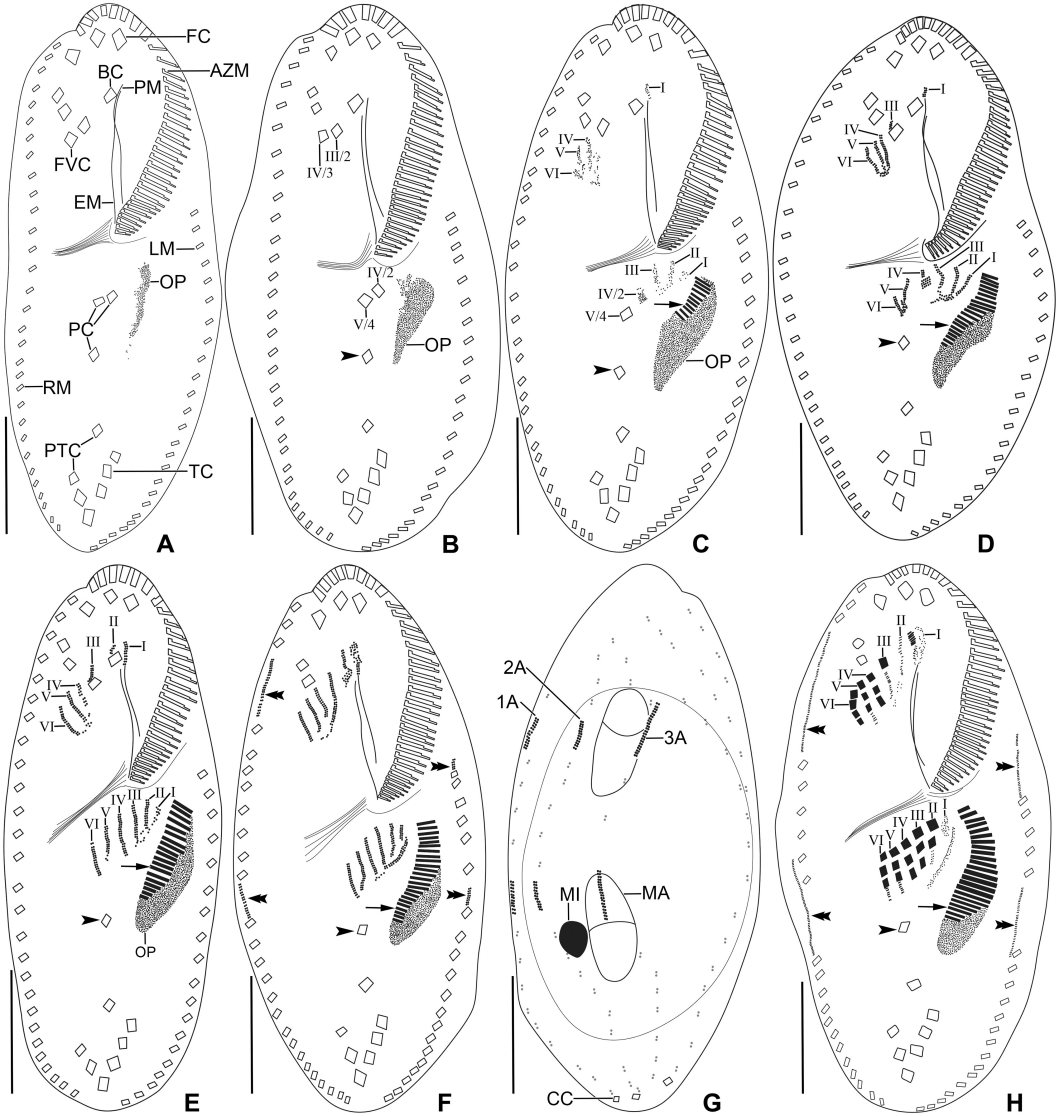
*Tetmemena polymorpha* n. sp. small morph dividers after protargol impregnation. Arrowheads mark the postoral cirrus V/3 which is not involved in anlagen formation, arrows show the new adoral membranelles, and double arrowheads indicate the marginal anlagen. **(A, B)** Ventral views of early dividers show the apokinetal origin of the oral primordium. **(C–E)** Ventral views of early dividers, showing (i) the dedifferentiating frontoventral cirrus IV/3 forms anlagen IV–VI of the proter, (ii) the anlagen I–III of the opisthe originate from the oral primordium, (iii) the cirrus IV/2 dedifferentiates to form anlage IV of the opisthe, (iv) the proliferation occurs anterior to paroral membrane, buccal cirrus, and cirrus III/2, and (v) the postoral cirrus V/4 dedifferentiates to form anlagen V and VI of the opisthe. **(F, G)** Ventral and dorsal view of an early divider, showing the formation of six frontal-ventral-transverse anlagen in the proter and opisthe, the marginal and dorsal kineties anlagen appear at two levels by within-row anlagen formation. The circle indicates dorsal bulge on dorsal side **(G)**. **(H)** Ventral view of a late mid-divider, showing the differentiation of anlagen into cirri and the extension of marginal anlagen. 1–3A, dorsal kineties anlagen; AZM, adoral zone of membranelles; BC, buccal cirrus; CC, caudal cirri; EM, endoral membrane; FC, frontal cirri; FVC, frontoventral cirri; I–VI, frontal-ventral-transverse anlagen; LM, left marginal row; MA, macronuclear nodule; MI, micronucleus; OP, oral primordium; PC, postoral cirri; PM, paroral membrane; PTC, pretransverse cirri; RM, right marginal row; TC, transverse cirri. Scale bars 20 μm.

**Figure 9:**
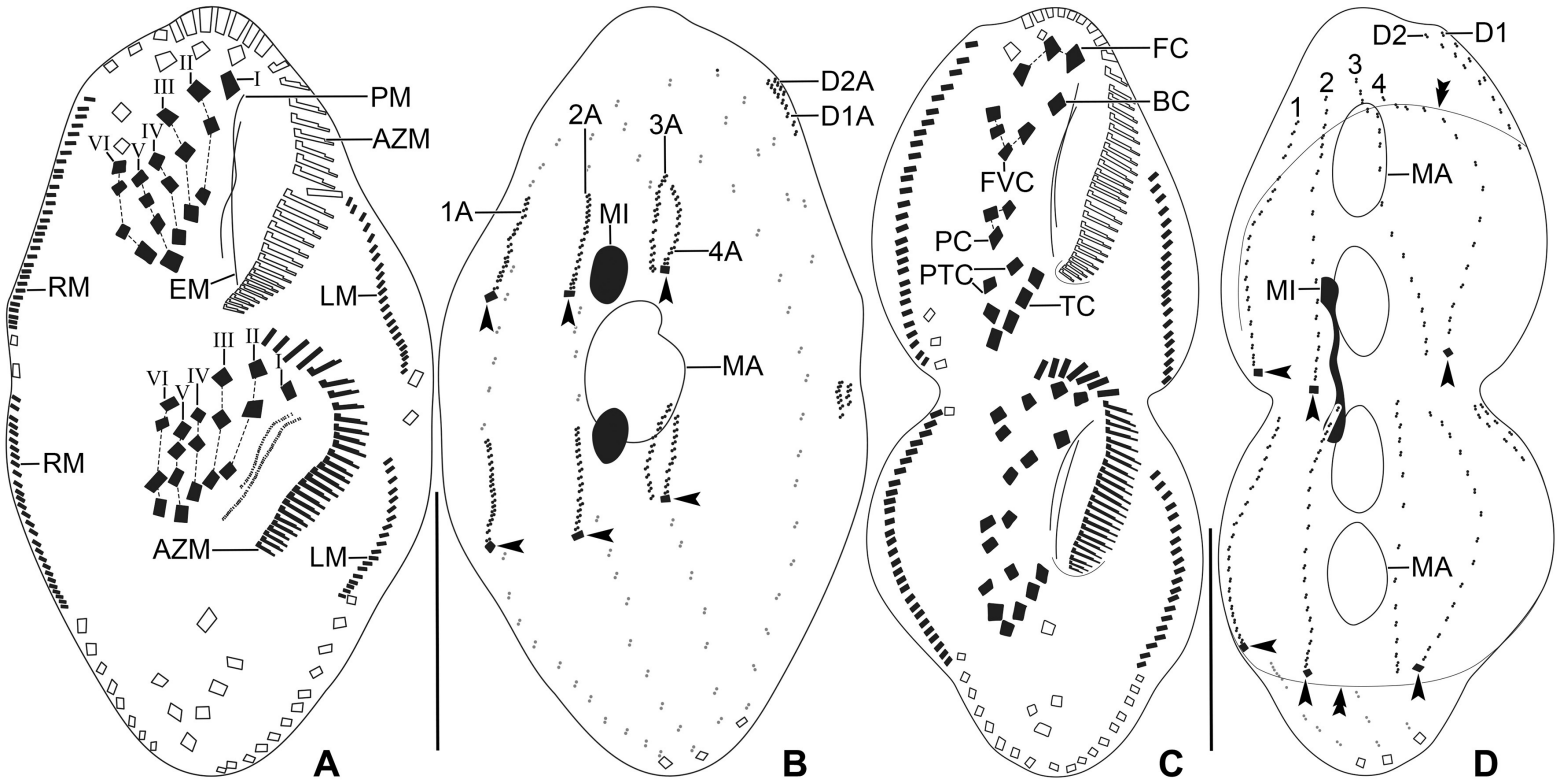
*Tetmemena polymorpha* n. sp. small morph dividers after protargol impregnation. **(A, B)** Ventral **(A)** and dorsal **(B)** view of a late divider, showing (i) the migrating new cirri to their final position, (ii) the complete new adoral zone and marginal cirri, (iii) the fragmentation in dorsal kinety 3 forming kinety 4, (iv) the new caudal cirri at ends of new kineties 1, 2, and 4 (arrowheads), and (v) the macronuclear nodules fuse into a single macronucleus. **(C, D)** Ventral **(C)** and dorsal **(D)** view of a late divider showing the frontal-ventral-transverse cirri at their final position. Arrowheads mark the new caudal cirri and double arrowheads indicate a bulge on the dorsal side. 1–4, dorsal kineties; AZM, adoral zone of membranelles; BC, buccal cirrus; EM, endoral membrane; FC, frontal cirri; FVC, frontoventral cirri; I–VI, frontal-ventral-transverse anlagen; LM, left marginal row; MA, macronucleus; MI, micronucleus; PC, postoral cirri; PM, paroral membrane; PTC, pretransverse cirri; RM, right marginal row; TC, transverse cirri. Scale bars 30 μm.

The three morphs of *Tetmemena polymorpha* n. sp. divide in the same ontogenetic pattern. Thus, dividers from the small morph are studied in detail (Figures 8A–H, 9A–D), and only three dividers and one postdivider of the giant morph are shown (Figures 7D–F). The ontogenetic mode is as described by Wirnsberger et al. (1985) for *T. pustulata* and *T. bifaria* and is characterized as follows: (1) the frontal-ventral-transverse cirral anlagen of the proter and opisthe originate separately; (2) the parental adoral zone is retained for the proter; (3) the undulating membranes anlage (anlage I) is partially reorganized; (4) the postoral cirrus V/3 is not involved in the anlagen formation; and (5) the simple fragmentation of dorsal kinety 3. Briefly, in the very early dividers, the oral primordium originates apokinetally as a longitudinal batch of basal bodies left to the postoral cirri and closer to the adoral zone of membranelles than to the transverse cirri. Then, the outline of this batch widens anteriorly and becomes obovate with disoriented anterior right margin. Three short streaks separate from the right anterior portion of the oral primordium to form frontal-ventral-transverse anlagen I–III of the opisthe (Figures 8A, B). Next, the new adoral membranelles start to develop in the right anterior portion of oral primordium. Simultaneously, cirrus IV/2 dedifferentiates to form anlage IV of the opisthe, and cirrus IV/3 disaggregates to form anlagen IV–VI of the proter. At the same time, the partial reorganization of the undulating membranes commences at the anterior end to form the proter's new left frontal cirrus (Figures 7D, 8C). Next, in late-early dividers, the postoral cirrus V/4 disaggregates and forms anlagen V and VI of the opisthe and cirri II/2 (buccal cirrus) and III/2 dedifferentiate and form streaks anteriorly to form anlagen II and III of the proter (Figures 8D–F). The six anlagen produce 18 cirri as in other oxytrichid species in the small morph; in large morph, anlage II rarely produces an extra buccal cirrus; and in the giant morph, anlagen II–IV usually produce one or two extra buccal cirri and one or two extra frontoventral cirri (Figures 7E, F, 8H, 9A, C).

The marginal anlagen appear at two levels by within-row anlagen formation. The anterior right marginal anlage arises by the disintegration of the fourth cirrus and elongates only posteriorly utilizing several parental cirri. The posterior right marginal anlage arises posterior to the mid-body and extends anteriorly right to the parental row and posteriorly within the row utilizing a few cirri. The anterior left marginal anlage appears anterior to the parental row and extends posteriorly utilizing few cirri and the posterior left marginal anlage arises posterior to the sixth or seventh cirrus and extends posteriorly utilizing few cirri (Figures 7E, 8F, H, 9A, C).

The dorsal ontogenesis is in *Oxytricha* pattern, i.e., anlagen arise “within-row” at two levels in kineties 1–3 in both the proter and opisthe in late-early dividers (Figure 8G). In middle stages of division, anlage 3 fragments in posterior region, forming anlage 4 in each daughter cell. Two dorsomarginal anlagen develop right of the anterior end of right marginal anlage in both proter and opisthe. A single caudal cirrus is formed at each posterior end of the new dorsal kineties 1, 2, and 4 (Figures 9B, D).

The nuclear division commences at very early dividers as the first sign of ontogenesis before the formation of the oral primordium when a replication band is formed in each macronuclear nodule and this stays unchanged until the mid-division process (Figure 8G). In mid-dividers, the two macronuclear nodules fuse to form a single macronucleus surrounded by micronuclei (Figure 9B). In late dividers, the macronucleus divides twice to form four nodules in very late dividers. Simultaneously, the micronuclei undergo mitotic division (Figure 9D).

### Occurrence and ecology

*Tetmemena polymorpha* n. sp. was previously recorded from freshwater canal in San Rossore, Pisa, Italy by Dini et al. (1975) and Ricci et al. (1975a) as “*Oxytricha bifaria*”. As mentioned in the Materials and Methods section, *Tetmemena polymorpha* n. sp. has been found in brackish water with a salinity of 4.7‰ and cultured in mineral water for several months. In the raw culture, the three morphs occur together. At that time, the difference between the SM and LM was unclear. To observe the growth of the cells and to differentiate between cells with different sizes in the culture, subcultures were established using single cells of different morphs from the clone culture. After a short lag phase, the exponential phase showed only cells of the LM, which divided very quickly and fed voraciously on yeast cells, starch grains, and other small ciliates (*Dexiostoma* sp. and *Tetrahymena* sp.) when added to the culture as food. In the stationary phase, most of the LM cells divided to produce mainly SM cells, which fed only on bacteria and yeast cells, and only very few LM cells divided to produce cannibalistic GM cells, which fed mainly on the SM cells and small ciliates when added to the culture as a food source. In some subcultures, the giant cells appeared only in the decline phase. In the decline phase, the LM cells gradually disappeared, while the SM cells increased in number and became dominant, while the GM cells increased in number but were always much fewer than the SM cells. The observations of several cultures showed that the three morphs are produced through cell division as follows: the LM cells produce and are produced by both SM and GM cells; the SM cells usually produce other SM cells or LM cells when extra food is added to the culture and never directly produce GM cells; the GM cells produce GM in the presence of only SM cells and bacteria as food in declining cultures, when adding extra food such as wheat grains, yeast, and small ciliates, they produce LM cells, and when starved, GM cells divide few times very quickly to produce LM and then SM cells. Resting cysts were obtained only from LM cells during the exponential phase by starvation. Several attempts were made to produce cysts from SM cells but all failed. Furthermore, we were unable to induce the excystment of the resting cysts under the lab conditions but we assume that they produce SM cells due to their small size (i.e., < 80 μm across *in vivo*). The SM and the LM show similar behavior, usually, they crawl slowly on the bottom of the culture dish and aggregate around the wheat grains and yeast, sometimes they swim slowly by rotating around the main body axis. The GM cells usually move very fast between SM cells or swim as other morphs, never resting. Conjugation occurred in the raw culture and never happened in the clone culture. No doublets were found in both raw and clone cultures.

### Phylogenetic analyses

**Figure 10:**
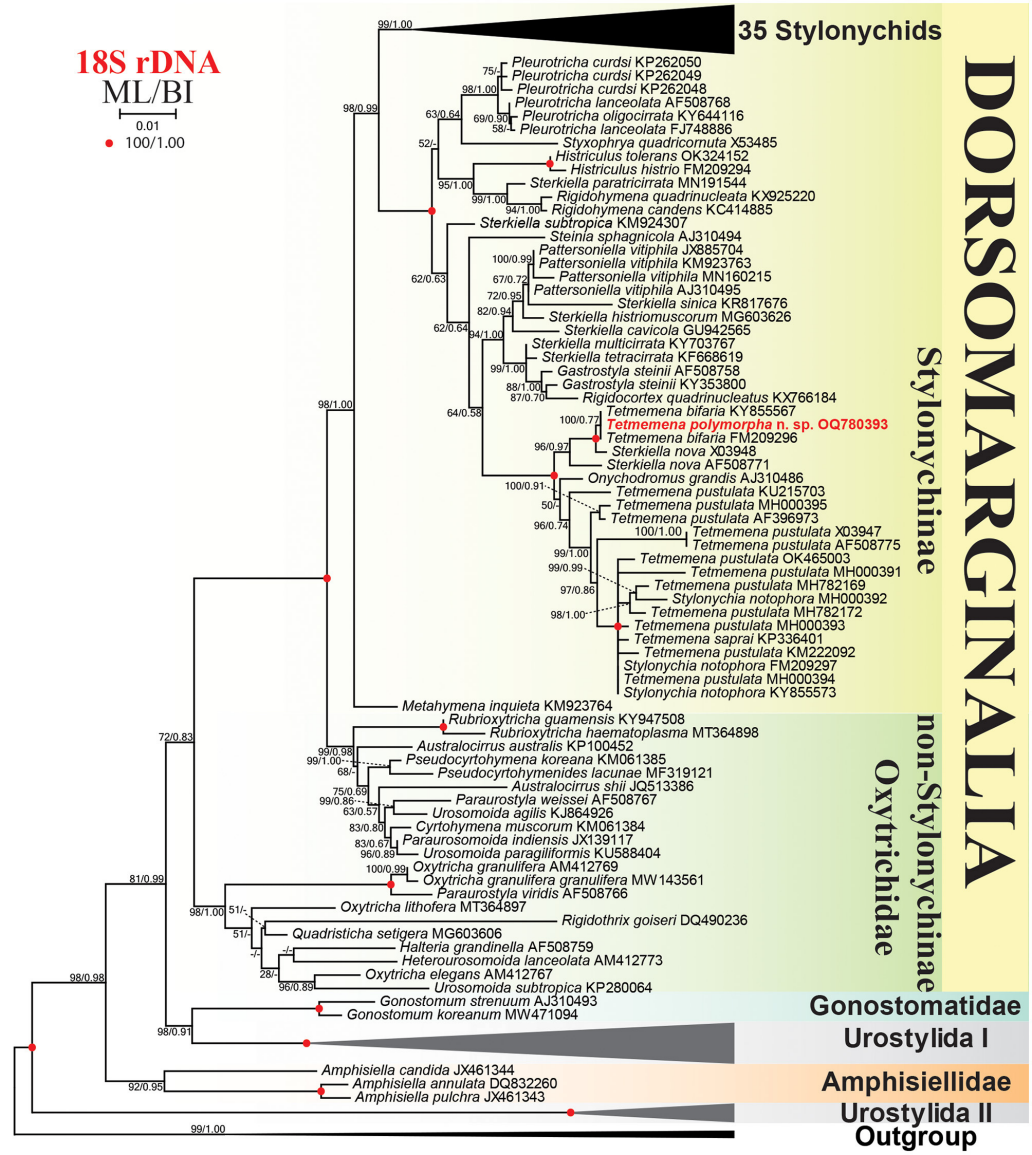
Maximum likelihood (ML) tree based on 18S rRNA gene sequences, showing the phylogenetic position of *Tetmemena polymorpha* n. sp. Newly obtained sequence is in bold. GenBank accession numbers follow species names. Numbers at the nodes represent the maximum likelihood bootstrap values and the Bayesian inference (BI) posterior probabilities. Dashes indicate bootstrap values < 50%, posterior probabilities < 0.5, or different topologies in BI and ML phylogenies. The scale bar represents one nucleotide substitution per 100 nucleotides.

The SSU rRNA gene sequence of *Tetmemena polymorpha* n. sp. is 1,622 base pairs long, has a GC content of 45.25%, and is available under GenBank accession number OQ780393. Phylogenetic trees constructed using ML and BI analyses show rather similar topologies, thus only the ML tree is presented with both the bootstraps (ML), and the posterior probabilities (BI) are included (Figure 10). The sequence of the new species is identical to two sequences identified as *Tetmemena bifaria* (KY855567, FM209296) in GenBank database. These three sequences form a clade together with two sequences of *Sterkiella nova* with full (100 ML, 1.00 BI; X03948) and high (96 ML, 0.97 BI; AF508771) supporting values. The subclade made by these five sequences is a sister to another subclade containing *Onychodromus grandis, Tetmemena* spp., and *Stylonychia notophora* sequences with full supporting values. The sequence of *Onychodromus grandis* (AJ310486) is placed as an adelphotaxon to the latter subclade with a low supporting value (50) in the ML and in a different position (i.e., forming a polytomy with this subclade and the subclade containing *Tetmemena polymorpha* n. sp. sequence) in the BI analysis. The 18S rRNA gene sequence of *T. polymorpha* n. sp. shows a similarity of 98.1–99.1% to other *Tetmemena* spp. and *S. notophora* sequences, 99.2% to *Onychodromus grandis* sequence, and 99.8 and 99.4% to the two *Sterkiella nova* sequences X03948 and AF508771, respectively.

## Discussion

### Generic assignment of *Tetmemena polymorpha* n. sp.

The genus *Tetmemena* was established to replace the junior homonym *Clara* Eigner, 1997 (Eigner, 1997, 1999). *Tetmemena* was diagnosed mainly based on the origin of the proter anlagen IV–VI, i.e., originate from frontoventral cirrus IV/3. At that time, Eigner (1999) included two species to the genus *Tetmemena*: *T. pustulata* (type species) and *Stylonychia vorax* Stokes, 1885. However, the latter was a misidentification of *Stylonychia bifaria* Stokes, 1887, so that it was transferred later to *Tetmemena* by Berger (2001) as *T. bifaria* (Stokes, 1887) Berger, 2001. Since then, two subspecies, *T. bifaria minima* Kumar et al., 2016 and *T. pustulata indica* Bharti et al., 2019, and one invalid species *Tetmemena saprai* Gupta et al., 2020, according to the International Commission on Zoological Nomenclature (ICZN) (2012, article 8.5.3), have been added. Eigner (1997) assigned the genus to the family Parakahliellidae Eigner, 1997, a synonym of the Kahliellidae according to Lynn (2008). However, Berger (1999) discussed this assignment in detail in his monograph on the Oxytrichidae. By combining data from the five taxa assigned to *Tetmemena*, the genus is characterized as follows: Stylonychinae, usually with 18 frontal-ventral-transverse cirri, transverse cirri in one or two groups, one left and one right marginal row, undulating membranes in *Stylonychia* pattern, four dorsal and two dorsomarginal kineties, dorsal kinety 3 with simple fragmentation, caudal cirri present, and anlagen IV–VI of the proter originate from the frontoventral cirrus IV/3. Morphologically and ontogenetically, the new species fits very well in the diagnosis of the genus *Tetmemena*.

The presence of giants was reported for very few oxytrichids, for instance, *Sterkiella cavicola*, which was recorded only one time by Maupas (1888) as “*Onychodromus grandis*” with a size of about 300 × 150 μm (Berger, 1999). The cannibalistic giants of *Sterkiella histriomuscorum* were recorded only by Giese and Aladen (1938). Furthermore, the giants of *Onychodromus grandis* and the cannibalistic giants of *Stylonychia curvata* were recorded in a single study by Tuffrau (1965). Dawson (1919) reported the production of cannibalistic individuals in an amicronucleate population of *Oxytricha hymenostoma*. Alonso and Perez-Silva (1963) recorded the giants of a species similar to *Stylonychia stylomuscorum* but with 2–4 micronuclei. The production of cannibalistic giants by *Styxophrya quadricornuta* was recorded in several studies (Lin and Prescott, 1985; Foissner et al., 1987; Wicklow, 1988; Kamra and Sapra, 1994). *Pattersoniella vitiphila* Foissner, 1987 produces normal and giant morphs but never showed cannibalistic behavior (Foissner, 1987). The giant formation is also found in other groups of ciliates such as the peniculine *Lembadion bullinum* (Kuhlmann, 1993; Kopp and Tollrian, 2003); the colpodeans *Colpoda cucullus* (Foissner, 1993), *C. maupasi* (Padnos, 1962), *Ottowphrya magna* (Foissner et al., 2002), and *Platyophryides latus* (de Puytorac et al., 1992); the tetrahymenids *Glaucoma ferox* (Foissner, 2013), *G. reniformis*, and *G. scintillans* (McCoy, 1975); and the heterotrichids *Blepharisma americanum, B. japonicum*, and *B. undulans* (Giese, 1938; Nilsson, 1967; Schorr and Boggs, 1974; Foissner and O'Donoghue, 1990). *Tetmemena polymorpha* n. sp. agrees with several of these species in that the cannibalistic giants appear only in the declining cultures as an adaptation to food depletion (Giese, 1938; Giese and Aladen, 1938; Foissner et al., 1987, 2002; Foissner and O'Donoghue, 1990). The seldom presence of this character in different groups of ciliates without reflecting phylogenetic relationships suggests that it is a result of converging evolution. Thus, at the present state of knowledge, we can consider it only as a species-specific character.

### Comparison of *Tetmemena polymorpha* n. sp. with related taxa

Members of *Tetmemena* could be divided into two groups, (i) *bifaria* group with transverse cirri separated into two groups and (ii) *pustulata* group with transverse cirri arranged in a single group. *Tetmemena polymorpha* n. sp. belongs to the *T. pustulata* complex and thus can be easily separated from *T. bifaria* by the arrangement of the transverse cirri, i.e., in one (vs. two) group (Wirnsberger et al., 1985; Berger, 1999; Kumar et al., 2016).

**Figure 11:**
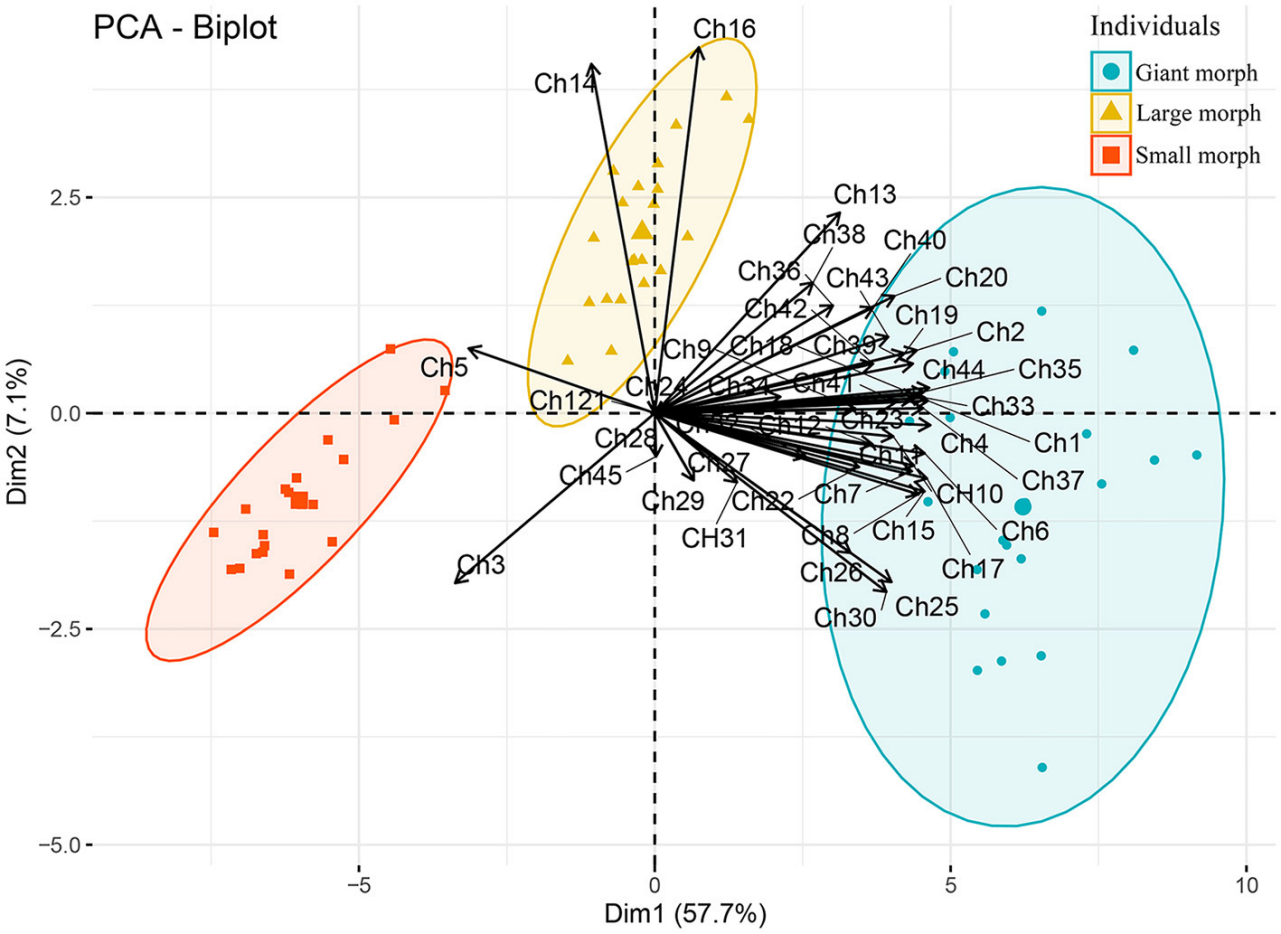
Plot of principal component analysis scores of standardized morphometric data. Solid circles, squares, and triangles each indicates a protargol-impregnated specimen. Characters (Ch1–Ch45) used for analysis are shown in Table 1.

Studying *T. polymorpha* n. sp. from a clone culture and during different stages of the culture using different techniques showed that it is a polymorphic species. The detailed morphometrics (Table 1) and the principal component analysis (Figure 11) also confirm these observations. The three morphs of *T. polymorpha* n. sp. distinctly differ from each other morphologically, thus observing them separately could lead to mistakenly assigning them to three distinct species.

The freshwater, cannibalistic giant forming population identified as “*Oxytricha bifaria*” and studied extensively (Esposito and Ricci, 1975; Ricci et al., 1975b, 1980, 1998; Esposito et al., 1976; Banchetti et al., 1978, 1982; Ricci, 1981, 1982) is morphologically identical to the new species (Ricci et al., 1985; Banchetti and Ricci, 1986; Rosati et al., 1988). Rosati et al. (1988) divided the specimens into normal and giant cells and both are smaller than the small and giant morphs of *T. polymorpha* n. sp., respectively. Furthermore, they did not mention the presence of large morph cells, very likely because they studied the morphology of a stock containing only small and giant cells (i.e., declining culture). However, the small and large morphs have rather similar morphology under low magnification and difficult to distinguish from each other without careful observation, comprehensive morphometric analysis, and studying the cells in different phases of the culture. No detailed description and morphometrics are provided in their work but their morphological data and SEM micrographs suggest that their population belongs to *Tetmemena polymorpha* n. sp. For instance, the body shape of both the normal (small) and giant morphs, the ventral cirral pattern, the dorsal ciliature, and the cyst morphology agree very well with those of the new species (Ricci et al., 1985; Rosati et al., 1988). Furthermore, the identical 18S rRNA gene sequences confirm their assignment to the same species (see below).

Berger (1999) preliminarily classified the Italian population as *T. pustulata* mainly because of the arrangement of the transverse cirri. *Tetmemena pustulata* is a very common freshwater stylonychid (Berger, 1999). It was studied several times, however, Berger (1999) suggests that the Austrian population of Wirnsberger et al. (1985) is authoritative. It is characterized mainly by the long dorsal kinety 4, i.e., as long as kinety 3, and the resting cyst possessing spines. The two clones studied by Wirnsberger et al. (1985) from the same population slightly differ from each other, but their morphometrics strongly overlap. They share some morphological characteristics with the SM of *T. polymorpha* n. sp. for instance, the body size (48–124 × 26–83 μm vs. 87–126 × 33–53 μm), the number of left marginal cirri (12–24 vs. 19–24), right marginal cirri (18–34 vs. 26–33), and adoral membranelles (24–42 vs. 28–41). The ventral cirral pattern of *T. pustulata*, 18 frontal-ventral-transverse cirri, is similar to that of both SM and LM of *T. polymorpha* n. sp. In contrast, *Tetmemena pustulata* has distinctly higher number (an average of 26 dikinetids in each of dorsal kineties 1–4) of dorsal dikinetids than the SM (on average 21, 20, 17, and 15 in dorsal kineties 1–4, respectively) and slightly lower than the LM (on average 35, 29, 24, and 25 in dorsal kineties 1–4, respectively) of *T. polymorpha* n. sp. Furthermore, the two species have similar resting cysts. *Tetmemena polymorpha* n. sp. can be distinguished from *T. pustulata* mainly by the presence (vs. absence) of cannibalistic giants. Moreover, both the LM and the GM of *T. polymorpha* n. sp. have larger body size (136–170 × 59–81 μm in LM and 200–232 × 90–132 μm in GM) and higher number of adoral membranelles (43–50 in LM and 62–83 in the GM) than *T. pustulata*. They also can be separated from each other by the dorsal kinety 4, i.e., distinctly shortened anteriorly in all morphs of *T. polymorpha* n. sp. while extending to the anterior body end in *T. pustulata*, an important, but highly underestimated, character in some stylonychine ciliates, for instance, it was found in *Stylonychia ammermanni* Gupta et al., 2001, *S. gibbera* Foissner, 2016, *S. koreana* Kumar et al., 2016, and both subspecies of *T. bifaria* (Berger, 1999; Gupta et al., 2001; Foissner, 2016; Kumar et al., 2016).

Several populations of *Tetmemena pustulata* have been studied since Wirnsberger et al. (1985). The only similar population to that of Wirnsberger et al. (1985) is the Chinese population studied by Shao et al. (2013). Other *T. pustulata* populations resemble the SM of *T. polymorpha* n. sp. but none of them produce large or giant specimens. For instance, the population studied by Foissner and Gschwind (1998) from Lake Mondsee in Austria, the same locality as the population of Wirnsberger et al. (1985), is very similar to the SM of *T. polymorpha* n. sp. and has a shortened dorsal kinety 4 but does not produce different morphs. Furthermore, the six Indian populations of *T. pustulata* studied by Kaur et al. (2020) have small body sizes, i.e., even smaller than the SM of *T. polymorpha* n. sp. (75–76 × 35–39 μm vs. 87–126 × 33–53 μm *in vivo*). Kaur et al. (2020) mentioned that the resting cysts have a smooth wall and a size of about 11.9 μm across. However, the scale bar on their figure shows that the cyst is about 26 μm across. The distinct differences between the Indian populations and the authoritative one from Wirnsberger et al. (1985), i.e., the shortened dorsal kinety 4 and the smooth resting cysts, indicate that the Indian populations represent distinct species.

The Indian *Tetmemena indica*, which was described as a subspecies of *T. pustulata* by Bharti et al. (2019), is a small species and thus can be compared only with the SM of *T. polymorpha* n. sp. It has a smaller body size (55–75 × 25–35 μm vs. 87–126 × 33–53 μm *in vivo*), fewer left and right marginal cirri (on average 10 and 20 vs. 22 and 30, respectively), lower total number of dorsal dikinetids (64–83 vs. 75–103), and lower number of dikinetids in kineties 3 and 4 (on average 13 each vs. 17 and 15, respectively). Furthermore, the resting cyst of *T. indica* has a smooth wall and separate macronuclear nodules (vs. with spines and fused macronucleus) (Bharti et al., 2019). It is also clear that *Tetmemena indica* distinctly differs from *T. pustulata* by several characteristics including the cell size, the number of marginal cirri and dorsal dikinetids, the length of the dorsal kinety 4, and most importantly, the shape of the resting cysts. Thus, they should be separated at the species level, i.e., *Tetmemena indica* Bharti et al., 2019 nov. stat. Foissner (2016) described a Venezuelan population of *T. pustulata* that is morphologically almost identical to *T. indica* but his description lacks cyst morphology. This population is unique within *T. pustulata* populations in having an emargination at the right posterior body end and thus possibly represents a distinct species.

*Tetmemena saprai* is also a small species, resembling the SM of *T. polymorpha* n. sp. in almost all morphometrics. It is characterized by the wavy outer layer of the resting cysts. Gupta et al. (2020) described the resting cysts as having three layers; however, from their micrograph, it is clear that they misinterpreted the wavy outer layer into two layers due to an incomplete focus.

*Stylonychia pseudograndis* Wang and Nie, 1935 resembles the GM of *T. polymorpha* n. sp. in several respects, i.e., it has a large body size (140–200 × 60–100 μm), high number of frontal, frontoventral, and buccal cirri (10–12), and connected macronuclear nodules. However, it differs from the GM of *T. polymorpha* n. sp. in the short adoral zone (about 43% of body length as calculated from the drawing vs. 51–68%), the left marginal row is J-shaped and the right row is slightly shortened posteriorly (vs. both rows J-shaped), the caudal cirri are distinctly (vs. slightly) shifted to right, and the transverse cirri are scarcely (vs. distinctly) projecting beyond the posterior end of the cell (Wang and Nie, 1935; Berger, 1999). Furthermore, neither small specimens nor cannibalistic behavior was recorded for *Stylonychia pseudograndis* (Wang and Nie, 1935).

### Phylogenetic analyses

The phylogenetic tree shows that *T. polymorpha* n. sp. clusters with two identical sequences. One of these sequences (FM209296) belongs to the Italian population of *T. polymorpha* n. sp. (viz., “*Oxytricha bifaria*”), i.e., it was provided to Schmidt et al. (2008) by G. Steinbrück (Universität Tübingen, Germany) who obtained the living cells of the Italian population from N. Ricci (University of Pisa, Italy) and studied the DNA in his laboratory (Schlegel, 1985; Schlegel and Steinbrück, 1986). The second sequence (KY855567), which was isolated from Kolleru Lake, India, lacks morphological description. However, since it is identical to the Italian sequence, very likely it was identified only based on the rRNA gene data. The two available *Sterkiella nova* sequences cluster with *T. polymorpha* n. sp. in the same subclade also lack morphological description (Elwood et al., 1985; Hewitt et al., 2003). Their location in the phylogenetic tree, i.e., distant from other *Sterkiella* spp., the lack of morphological description, and the high morphological and ontogenetic similarity between *Sterkiella nova* and members of *Tetmemena pustulata* complex (Foissner and Berger, 1999), suggest the possibility of misidentification. The placement of *Onychodromus grandis* within the subclade containing other *Tetmemena* sequences seems justified because they have rather similar ontogenesis (Szabó and Wilbert, 1995).

The second subclade contains another 16 sequences, three *Stylonychia notophora*, one *T. saprai*, and 12 *T. pustulata*. The *Stylonychia notophora* sequences lack morphological identification, two of them are identical to a sequence of an Indian population (MH000394) of *T. pustulata* and the third sequence of *S. notophora* shows a similarity of 99.5% to and clusters with another Indian sequence (MH782169) of *T. pustulata* (Kaur et al., 2020). These data suggest that these sequences very likely belong to the *Tetmemena pustulata* complex. The invalid species *Tetmemena saprai*, which is a distinct species mainly based on the cyst morphology (Gupta et al., 2020), shows a similarity of 99.9% to each of the Indian *T. pustulata* population (MH000394) (Kaur et al., 2020) and the two *S. notophora* (FM209297 and KY855573) sequences. Within the 12 *T. pustulata* sequences, only the six Indian populations studied by Kaur et al. (2020) are characterized morphologically. Interestingly, the morphometric characters of these populations are identical to each other but their rRNA gene sequences are different (i.e., showing 98.9–99.9% similarity among the six populations) and scattered within the subclade. Taking into account that sequences from the same stylonychid species are usually identical, e.g., the sequence of *T. polymorpha* n. sp. is identical to the one from the Italian population and to another sequence, most likely belonging to the same species, from India. The other example is *T. saprai*, which shows only 0.1% dissimilarity from the most similar species although they are morphologically different (Gupta et al., 2020). Thus, the genetic data of the Indian *T. pustulata* seems inconsistent with the morphological data. However, Kaur et al. (2020) did not mention whether the cyst they described belongs to a single population or all populations produce cysts with the same morphology, an important characteristic used for the differentiation between morphologically similar species (Kumar et al., 2016; Bharti et al., 2019; Omar et al., 2022). Within the 12 sequences of *T. pustulata* in GenBank database, only two of which are identical to each other indicating that *T. pustulata* complex contains a high number of cryptic or pseudocryptic species. Therefore, morphological identification based on important features, such as the length and number of dikinetids of individual dorsal kineties and the resting cyst morphology for each population, and a ribosomal RNA gene sequence of *T. pustulata* representing the authoritative population of Wirnsberger et al. (1985), i.e., with dorsal kinety 4 as long as kinety 3 and with resting cyst possessing spines, are necessary to solve this issue.

## Data availability statement

The datasets presented in this study can be found in online repositories. The names of the repository/repositories and accession number(s) can be found in the article/Supplementary material.

## Author contributions

AO and J-HJ designed the study and revised the manuscript. AO performed morphological experiments, molecular experiments, and data analyses. AO, SJ, and J-HJ wrote the manuscript. All authors read and approved the final version of the manuscript.
