# Supplementary material for: Polymorphism, gigantism, and cannibalism, one stylonychid ciliate (Ciliophora, Hypotricha) to rule them all
Source: Front Microbiol. 2023 May 17;14:1159634. doi: 10.3389/fmicb.2023.1159634 (PMC10229871; doi:10.3389/fmicb.2023.1159634)
Supplement: Supplementary file 1 [file Image_1.pdf]

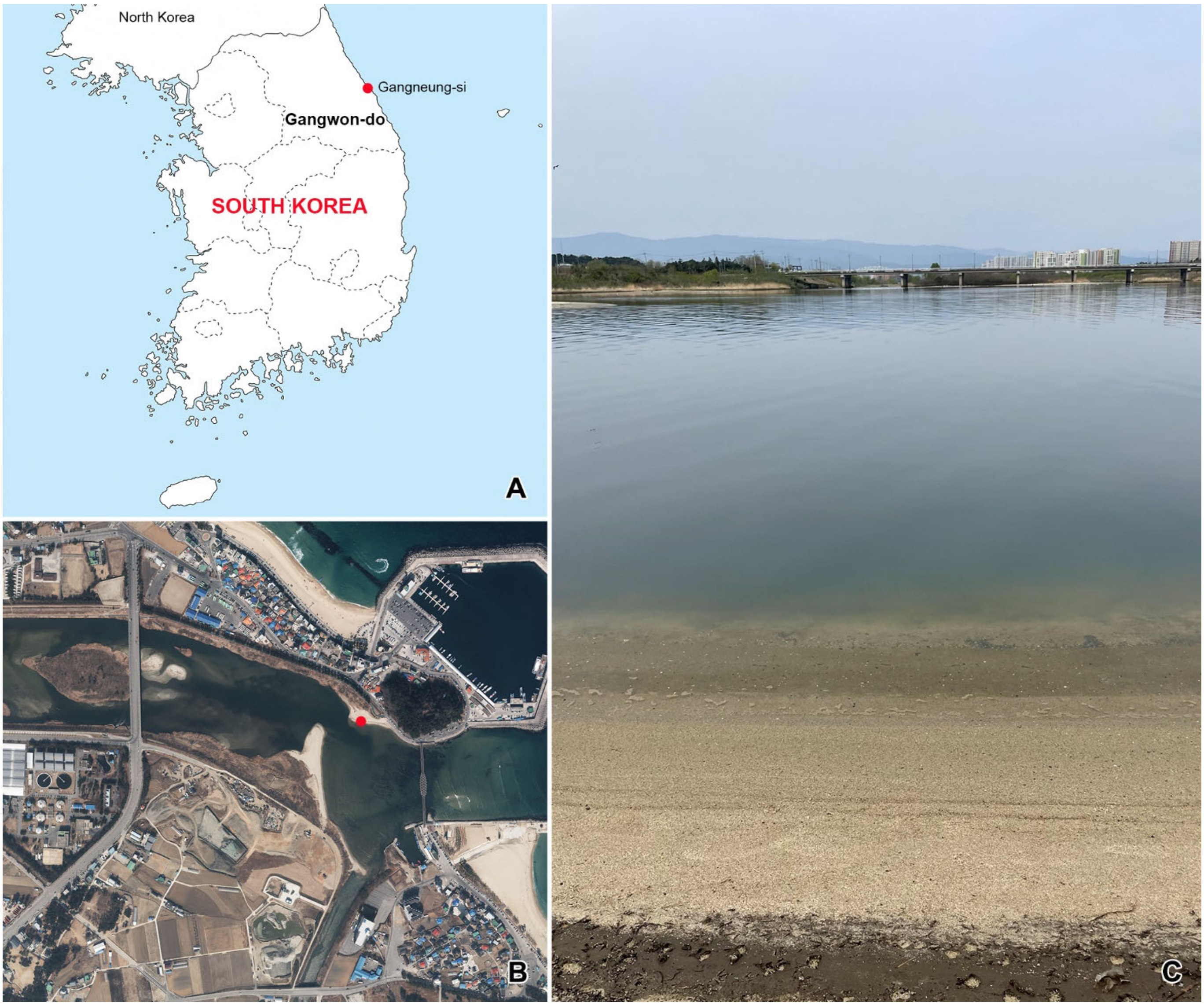

Supplementary Figure 1 | Sample location. (A) Map of South Korea and the sampling site in Gangneung-si. (B, C) The estuary of Namdaecheon Stream.
